# Decreased *RYR2* Cluster Size and Abnormal SR Ca^2+^ Release Contribute to Arrhythmogenesis in *TMEM43*‐Related ARVC

**DOI:** 10.1002/advs.202512058

**Published:** 2025-09-15

**Authors:** Jiaxi Shen, Xiaochen Wang, Hangping Fan, Yaxun Sun, Tingyu Gong, Hangyuan Qiu, Jue Wang, Ziwei Pan, Yanna Dang, Hongkun Wang, Danni Zhou, Tianyi Zhu, Hao Wang, Xianzhen Chen, Lizhen Xu, Jun Su, Fan Yang, Yiquan Tang, Xuguang Li, Bing Yang, Lenan Zhuang, Wei Wang, Chenyang Jiang, Ping Liang

**Affiliations:** ^1^ Key Laboratory of Combined Multi‐Organ Transplantation Ministry of Public Health The First Affiliated Hospital Zhejiang University School of Medicine Hangzhou 310003 China; ^2^ Institute of Translational Medicine Zhejiang University Hangzhou 310029 China; ^3^ Key Laboratory of Precision Prevention and Treatment for Atherosclerotic Diseases of Zhejiang Province Department of Cardiology The First Affiliated Hospital of Ningbo University Ningbo 315010 China; ^4^ Department of Cardiology Sir Run Run Shaw Hospital Zhejiang University School of Medicine Hangzhou 310016 China; ^5^ Shulan International Medical College Zhejiang Shuren University Hangzhou 310015 China; ^6^ Department of Veterinary Medicine College of Animal Sciences Zhejiang University Hangzhou 310058 China; ^7^ Zhejiang Provincial Key Laboratory for Cancer Molecular Cell Biology Life Sciences Institute Zhejiang University Hangzhou 310058 China; ^8^ Prenatal Diagnosis Center Hangzhou Women's Hospital Hangzhou 310008 China; ^9^ Department of Dermatology and Venereology Sir Run Run Shaw Hospital Zhejiang University School of Medicine Hangzhou 310016 China; ^10^ Department of Biophysics and Kidney Disease Center of the First Affiliated Hospital Zhejiang University School of Medicine Hangzhou 310058 China; ^11^ State Key Laboratory of Medical Neurobiology and MOE Frontiers Center for Brain Science Institutes of Brain Science Fudan University Shanghai 200032 China; ^12^ Department of Cardiology Shanghai General Hospital School of Medicine Shanghai Jiao Tong University Shanghai 200080 China; ^13^ Cardiovascular Research Institute of Jiangxi Province Jiangxi Provincial People's Hospital The First Affiliated Hospital of Nanchang Medical College Nanchang 330006 China

**Keywords:** arrhythmia, ARVC, iPSC‐CMs, lamin B2, *RYR2*, *TMEM43*

## Abstract

Arrhythmogenic right ventricular cardiomyopathy (ARVC) is a rare inherited cardiomyopathy featured by life‐threatening arrhythmias. While *TMEM43* has been identified as an ARVC‐associated gene, molecular links between *TMEM43* mutations and electrophysiological abnormalities in ARVC remain largely elusive. Here, using induced‐pluripotent‐stem‐cell‐derived cardiomyocytes (iPSC‐CMs) and knock‐in mice as models, it is demonstrated that a novel *TMEM43* mutation (TMEM43‐P386S) causes Ca^2+^ dysregulation that leads to arrhythmic phenotypes in ARVC, which can be prevented by flecainide. Mechanistically, *TMEM43* interacts with lamin B2, and the TMEM43‐P386S mutation induces lamin B2 mislocalization and abnormal nuclear envelope structure in ARVC iPSC‐CMs, resulting in decreased chromatin opening of promoters associated with downregulated genes, including ryanodine receptor 2 (*RYR2*). *RYR2*s are downregulated and grouped into smaller clusters in ARVC iPSC‐CMs, as revealed by Tau‐STED super‐resolution imaging, contributing to enhanced *RYR2*‐mediated sarcoplasmic reticulum Ca^2+^ leak. These findings represent a novel mechanism underlying arrhythmogenesis in *TMEM43*‐related ARVC and point to *RYR2* stabilization as a potential therapeutic strategy.

## Graphical abstract

The TMEM43 ‐ P386S mutation causes arrhythmogenic right ventricular cardiomyopathy (ARVC) by mislocalizing itself from nuclear envelope (NE) to cytoplasm, disrupting lamin B2 (a novel *TMEM43* interactor) localization, NE integrity and chromatin accessibility, causing hyper ‐ phosphorylation and reduced expression/clustering of ryanodine receptor type 2 (*RYR2)*, enhanced sarcoplasmic reticulum (SR) calcium leak and arrhythmias, which can be rescued by flecainide or KN93.

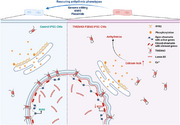

## 1 Introduction

Arrhythmogenic right ventricular cardiomyopathy (ARVC) is a rare inherited cardiomyopathy featured by life‐threatening arrhythmias, heart failure, and sudden cardiac death (SCD).^[^ ^1^ ^]^ The pathological characteristics of ARVC is that the right ventricular myocytes are gradually replaced by adipose tissue or fibrous adipose tissue, resulting in diffuse expansion of right ventricle and weakening of contractile movement.^[^ ^1^ ^]^ These changes may not be apparent in the early stage of the disease, during which patients may still be at risk of ventricular arrhythmias, leading to diagnostic difficulties and uncertainties. ARVC is one of the major causes of SCD in young adults and athletes, and strenuous exercise may trigger malignant ventricular arrhythmias and increase the risk of SCD.^[^ ^2^ ^]^

At least 16 genes associated with ARVC have been identified so far, encoding desmosome, adherens junction, cytoskeletal structure, ion transport, and cytokine proteins, respectively.^[^ ^3^, ^4^ ^]^ Mutations in desmosomal genes were identified in approximately half of patients with ARVC.^[^ ^1^, ^3^, ^4^ ^]^ Cytoskeletal structure proteins, including desmin, lamin A, transmembrane protein 43 (*TMEM43*), titin, and filamin C, constitute the second‐largest category of ARVC‐associated mutations.^[^ ^4^ ^]^

*TMEM43* (encoded by the *TMEM43* gene), first identified in 2001, is highly conserved across species and is present in mammalian heart.^[^ ^5^, ^6^ ^]^ The function of *TMEM43* is poorly understood, although it has been evidenced that *TMEM43* is associated with linker of nucleoskeleton and cytoskeleton (LINC) complex components for physiological maintenance of the nuclear structure.^[^ ^7^, ^8^ ^]^ Missense mutations in the *TMEM43* gene cause ARVC type 5 (ARVC5).^[^ ^9^ ^]^ However, the precise mechanisms linking the *TMEM43* mutations to increased arrhythmogenesis are largely elusive.

Here, we identified a novel *TMEM43* mutation (c.1156C>T, p.P386S) in a patient who was clinically diagnosed with ARVC. We employed patient‐specific and genome‐edited human induced‐pluripotent‐stem‐cell‐derived cardiomyocytes (iPSC‐CMs), as well as TMEM43‐P386S knock‐in mice to investigate both in vitro and in vivo phenotypes. This comprehensive approach allowed us successfully uncover the pathological characteristics associated with *TMEM43*‐related ARVC and to elucidate the molecular mechanisms underlying the increased propensity for cardiac arrhythmias observed in *TMEM43*‐related ARVC.

## 2 Results

### 2.1 Clinical Characteristics

We recruited a family pedigree with one clinically diagnosed ARVC patient who is a 51 years old male (Figure S1A, Supporting Information). The patient presented with paroxysmal palpitation and syncope in March 2016. The electrocardiogram (ECG) in emergency room showed ventricular tachycardia (VT) with left bundle branch block morphology, and the rate of VT was 176 bpm. Cardioversion was applied to terminate the VT. After the restoration of sinus rhythm, the inverted T wave on V1–V3 lead and epsilon wave on V1, V2 lead could be observed on the 12‐lead ECG (Figure S1B, Supporting Information). The F1 lead on Fontaine pattern showed dubious epsilon wave. Enlarged right ventricle and obvious aneurysm was detected on the echocardiography and enhanced cardiac magnetic resonance imaging (MRI) (Figure S1C, Supporting Information). Right ventricular ejection fraction on cardiac MRI was 12%, and left ventricular ejection fraction was within normal range. A dual chamber implantable cardioverter defibrillator (ICD) was implanted for secondary prevention. VT episodes recurred twice in April 2016 and May 2016, and both episodes were terminated by ICD shocks. VT ablation was performed in Aug 2021. Large low‐voltage area was mapped and ablated by high‐density epicardial substrate mapping in right ventricular outflow tract. Late abnormal ventricular activities were recorded and completely ablated (Figure S1D, Supporting Information). Genetic testing revealed a heterozygous mutation (c.1156C>T, p.P386S) in the *TMEM43* gene (TMEM43‐P386S) (Figure S1E, Supporting Information), which has not been previously described. The location of this mutation is within the transmembrane domain of *TMEM43*, which is highly conservative across species (Figure S1F, Supporting Information). The pathogenicity of the mutation was classified as “likely pathogenic” according to the American College of Medical Genetics and Genomics guideline.^[^ ^10^ ^]^ No other abnormalities were detected in the other genes screened that have been associated with ARVC. The daughter of the patient reported 3 years history of polymorphic VT. No history of sudden death or syncope was reported in this family. No arrhythmic event was recorded by ICD of the proband in 6 months post the ablation procedure.

### 2.2 Generation and Characterization of Patient‐Specific iPSC‐CMs

**Figure 1:**
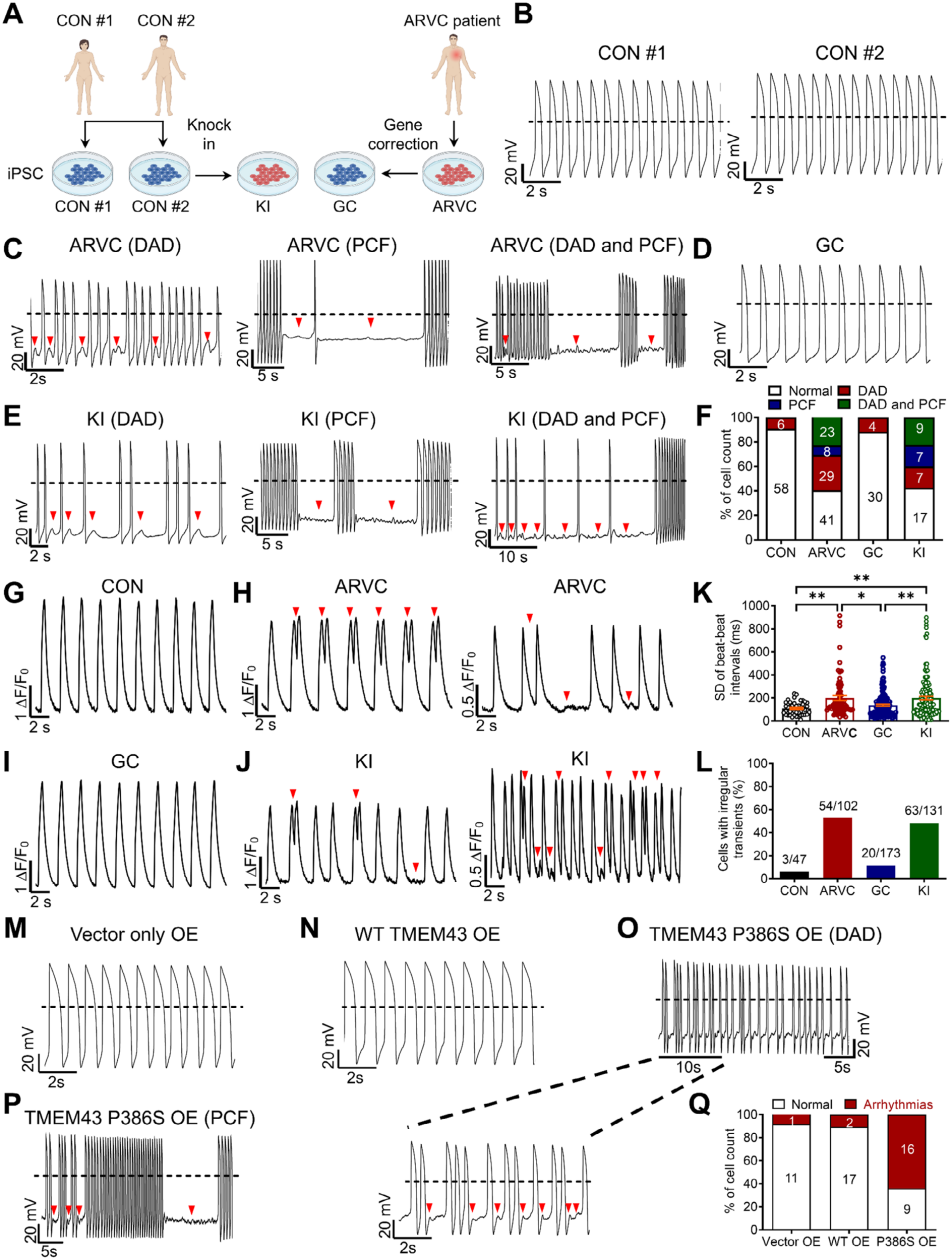
TMEM43‐P386S causes arrhythmic phenotypes in ARVC iPSC‐CMs. A) Cartoon to show the control, patient‐specific, and genome‐edited iPSC lines generated in this study. B–E) Representative action potential tracings recorded by single‐cell patch clamp from control (CON #1 and CON #2), ARVC patient‐specific (ARVC), gene‐corrected (GC), and TMEM43‐P386S knock‐in (KI) iPSC‐CMs. Dashed lines indicate 0 mV. Red arrows indicate the delayed afterdepolarization (DAD) or paroxysmal cellular flutter (PCF) arrhythmias. F) Bar graph to compare the percentage of cells with arrhythmias between control (*n* = 64), ARVC (*n* = 101), GC (*n* = 34) and KI (*n* = 40) iPSC‐CMs. Data were collected from 2 different iPSC lines. G–J) Representative Ca^2+^ transient tracings recorded by Fluo‐4 Ca^2+^ imaging from control (CON), ARVC, GC, and KI iPSC‐CMs. Red arrows indicate the irregular arrhythmia‐like Ca^2+^ transients. K) Bar graph to compare the SD of beat–beat intervals between control (*n* = 47), ARVC (*n* = 72), GC (*n* = 154), and KI (*n* = 131) iPSC‐CMs. Data were collected from 2 different iPSC lines. L) Bar graph to compare the percentage of cells with irregular transients between control (*n* = 47), ARVC (*n* = 102), GC (*n* = 173), and KI (*n* = 131) iPSC‐CMs. Data were collected from 2 different iPSC lines. M–P) Representative action potential tracings recorded by single‐cell patch clamp from control iPSC‐CMs overexpressing lentiviral‐GFP (Vector only OE), lentiviral‐WT TMEM43‐GFP (WT *TMEM43* OE), and lentiviral‐TMEM43‐P386S‐GFP (TMEM43‐P386S OE). Dashed lines indicate 0 mV. Red arrows indicate the DAD or PCF arrhythmias. Q) Bar graph to compare the percentage of cells with arrhythmias between Vector only OE, WT *TMEM43* OE, and TMEM43‐P386S OE groups. *n* = 12–25 cells. For all panels, data are represented as mean ± SEM. ^*^ *p* < 0.05; ^**^ *p* < 0.01, one‐way ANOVA followed by Tukey's honestly significant difference (HSD) post‐hoc test (K).

A skin biopsy was obtained from the ARVC patient and skin fibroblasts were reprogrammed by nonintegrated Sendai virus (**Figure** **1**A; Figure S2A, Supporting Information). The generated patient‐specific iPSCs exhibited typical human embryonic stem cell morphology (Figure S2B, Supporting Information), normal karyotype (Figure S2C, Supporting Information), and alkaline phosphatase (ALP) activity (Figure S2D, Supporting Information), stained positive for pluripotent markers (Figure S2E), and gave rise to derivatives from all three germ layers of endoderm, mesoderm, and ectoderm (Figure S2F, Supporting Information). As controls, we used iPSC lines that were characterized in previous studies (Figure 1A; Table S1, Supporting Information).^[^ ^11^ ^]^ Sanger sequencing confirmed the existence of the *TMEM43* missense mutation in ARVC but absence in control at the iPSC level (Figure S2G, Supporting Information). A small molecule‐based 2D monolayer cardiac differentiation protocol was applied as described previously.^[^ ^11^, ^12^ ^]^ Around day 10 after the cardiac differentiation, differentiated cells demonstrated cardiac morphology with spontaneous beating and positive staining of cardiac‐specific markers *TNNT2* and α‐actinin (Figure S2H, Supporting Information).

### 2.3 TMEM43‐P386S Causes Arrhythmic Phenotypes in ARVC iPSC‐CMs

Lethal ventricular tachyarrhythmia is a clinical feature of ARVC.^[^ ^13^, ^14^ ^]^ We therefore investigated the electrophysiological properties of ARVC iPSC‐CMs carrying TMEM43‐P386S by single‐cell patch clamp recordings. Control iPSC‐CMs exhibited uniform and rhythmic action potential waveforms (Figure 1B–F). By contrast, a large subfraction of ARVC iPSC‐CMs (60%, *n* = 101) as compared to controls (9%, *n* = 64) were observed to exhibit arrhythmic waveforms, mainly manifesting as three distinct phenotypes: 1) delayed afterdepolarization (DAD) (control: 9%; ARVC: 29%); 2) paroxysmal cellular flutter (PCF) (control: 0%; ARVC: 8%); 3) both DAD and PCF (control: 0%; ARVC: 23%) (Figure 1C–F). Moreover, analysis of key parameters of action potentials revealed significantly increased peak–peak interval variability, and reduced maximal diastolic potential (MDP), action potential amplitude (APA), and maximal upstroke velocity (*V*_max_) in ARVC iPSC‐CMs compared to their control counterparts (Table S2, Supporting Information).

To rule out the individual variability between iPSC lines from different healthy controls, we generated isogenic control lines by CRSIPR/Cas9‐mediated genome editing technology (Figure 1A; and Figure S3, Supporting Information). The resultant gene‐corrected (GC) iPSC‐CMs showed dramatic reduction of arrhythmic incidence (12%, *n* = 34), resembling the action potential profile of control iPSC‐CMs (Figure 1D–F; Table S2, Supporting Information). To ensure the pathogenicity of TMEM43‐P386S, we introduced the P386S mutation in the healthy control iPSC line (control #2) to generate knock‐in (KI) iPSC lines (Figure 1A). Importantly, recordings of action potentials revealed the arrhythmic phenotypes in KI iPSC‐CMs (Figure 1E,F; Table S2, Supporting Information). Consistently, Fluo‐4 Ca^2+^ imaging data also revealed a significantly increased incidence of irregular Ca^2+^ transients in ARVC and KI iPSC‐CMs, which was virtually absent in control and GC iPSC‐CMs (Figure 1G–L). In addition, we constructed a lentiviral plasmid with mutant *TMEM43*, in which the P386S mutation was introduced. Wildtype (WT) and mutant constructs were subsequently subcloned into the cop‐green‐fluorescent‐protein (GFP)‐carrying vector (pCDH‐CMV‐MCS‐EF1‐copGFP) (Figure S4, Supporting Information). Control iPSC‐CMs overexpressing vector alone or WT *TMEM43* showed a uniform action potential pattern (Figure 1M–Q). However, iPSC‐CMs overexpressing mutant *TMEM43* were detected to recapitulate the arrhythmic phenotypes similar to ARVC iPSC‐CMs (Figure 1O–Q). Taken together, these results demonstrate that TMEM43‐P386S causes arrhythmic phenotypes in ARVC iPSC‐CMs.

### 2.4 β‐Adrenergic Challenge Exacerbates Arrhythmic Phenotypes in ARVC iPSC‐CMs

**Figure 2:**
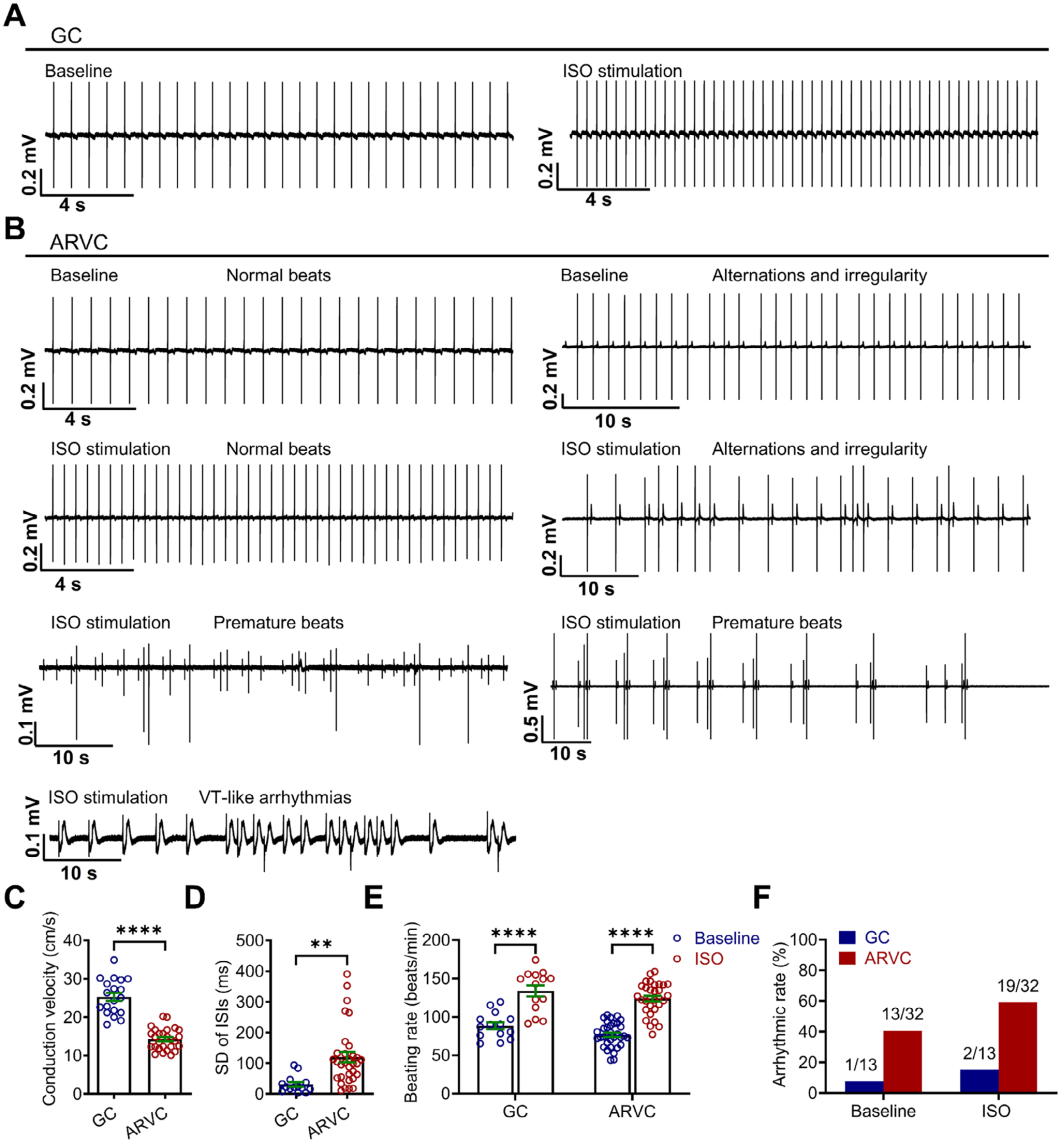
β‐adrenergic challenge exacerbates arrhythmic phenotypes in ARVC iPSC‐CMs. A) Representative field potential tracings recorded from GC iPSC‐CMs at baseline and 1 µm isoproterenol (ISO) stimulation. B) Representative field potential tracings recorded from ARVC iPSC‐CMs at baseline and 1 µm ISO stimulation. Less severe arrhythmias such as alternations and irregularity were observed in baseline ARVC iPSC‐CMs. More severe arrhythmias, including premature beats and VT‐like arrhythmias, were observed in ISO‐treated ARVC iPSC‐CMs. Red arrows indicate the arrhythmogenic activities. C–E) Bar graphs to compare the conduction velocity, SD of interspike intervals (ISIs), and beating rate between GC and ARVC iPSC‐CMs. *n* = 14–33 independent MEA experiments. Data were collected from 2 different iPSC lines. F) Bar graph to compare the arrhythmic rate between GC (*n* = 13 independent MEA experiments) and ARVC (*n* = 32 independent MEA experiments) iPSC‐CMs at baseline and 1 µm ISO stimulation, respectively. Data were collected from 2 different iPSC lines. For all panels, data are represented as mean ± SEM. ^**^ *p* < 0.01; ^****^ *p* < 0.0001, unpaired two‐tailed Student's *t*‐test (C–E).

The arrhythmic phenotypes of ARVC iPSC‐CMs were also identified at the multicellular level by multielectrode array (MEA), which were manifested as alternations and irregularity, and were observed in 40.6% (13 of 32 independent MEA studies) of ARVC iPSC‐CMs, but in 7.7% (1 of 13 independent MEA studies) of GC iPSC‐CMs (**Figure** **2**A–F). In addition, ARVC iPSC‐CMs showed significantly reduced conduction velocity and increased beat interval variability as compared to GC iPSC‐CMs (Figure 2C,D). Given the reduced *V*_max_ and conduction velocity in ARVC iPSC‐CMs, endogenous sodium currents were isolated from GC and ARVC iPSC‐CMs. Notably, ARVC iPSC‐CMs exhibited significantly reduced sodium current density, with steady‐state activation curve being right‐shifted and steady‐state inactivation curve being left‐shifted (Figure S5A–E, Supporting Information). The L‐type calcium currents and potassium currents were comparable between GC and ARVC iPSC‐CMs (Figure S5F–O, Supporting Information).

Clinically, isoproterenol (ISO) testing is a viable tool for early diagnosis of ARVC.^[^ ^15^, ^16^ ^]^ We next assessed whether ISO application exaggerates the arrhythmic phenotypes in ARVC iPSC‐CMs. Beating rate was markedly accelerated in both GC and ARVC iPSC‐CMs upon treatment of ISO (1 µm) (Figure 2E). Notably, although ISO‐stimulated GC iPSC‐CMs exhibited a higher arrhythmic rate (15.4%) than baseline, only less severe arrhythmias such as alternations and irregularity were observed (Figure 2A,F). By contrast, ISO stimulation in ARVC iPSC‐CMs increased the arrhythmic rate to 59.4% and elicited more severe arrhythmias including premature beats and VT‐like arrhythmias, which were absent at baseline (Figure 2B,F). These results suggest that ARVC iPSC‐CMs are more susceptible to arrhythmias on β‐adrenergic stimulation.

### 2.5 Transcriptomic Analysis of ARVC iPSC‐CMs

Having determined that P386S is a pathogenic mutation that causes *TMEM43*‐related ARVC, we further identified potential signaling pathways closely associated with the arrhythmic phenotypes by performing genome‐wide RNA‐sequencing (RNA‐Seq) to compare the transcriptomes of ARVC iPSC‐CMs with their isogenic controls (Figure S6A, Supporting Information). 2698 out of 28 137 total genes were differentially expressed in ARVC iPSC‐CMs, in which 1751 genes were upregulated and 947 genes were downregulated (Figure S6B,C, Supporting Information). We found that Ca^2+^ signaling pathway (hsa04020) was among the top ten enriched Kyoto Encyclopedia of Genes and Genomes (KEGG) pathways (Figure S6D, Supporting Information). In addition, there were multiple Ca^2+^‐related Gene Ontology (GO) terms enriched in the differentially expressed genes (DEGs) (Figure S6E, Supporting Information). Interestingly, transcripts of several major Ca^2+^‐handling proteins were downregulated in ARVC iPSC‐CMs (Figure S7A, Supporting Information). Western blot analysis revealed upregulation of calcium–calmodulin (CaM)‐dependent protein kinase II (*CaMKII*) phosphorylation level, downregulation of sarcoplasmic reticulum calcium ATPase 2a (*SERCA2a*), and ryanodine receptor 2 (*RYR2*) expression levels, whereas the expressions of voltage‐gated calcium channel 1.2 (Ca_v_1.2), sodium–calcium exchanger 1 (NCX1), and phospholamban (*PLN*) (total and phosphorylated) were unaltered in ARVC iPSC‐CMs compared with GC iPSC‐CMs (Figures S7B–D and S8, Supporting Information). The protein levels of voltage‐gated sodium channel 1.5 (Na_v_1.5) and gap junction protein connexin 43 were comparable between GC and ARVC iPSC‐CMs (Figure S8, Supporting Information).

### 2.6 Abnormal Sarcoplasmic Reticulum Ca^2+^ Release Is Causally Linked to the Arrhythmic Phenotypes in ARVC iPSC‐CMs

**Figure 3:**
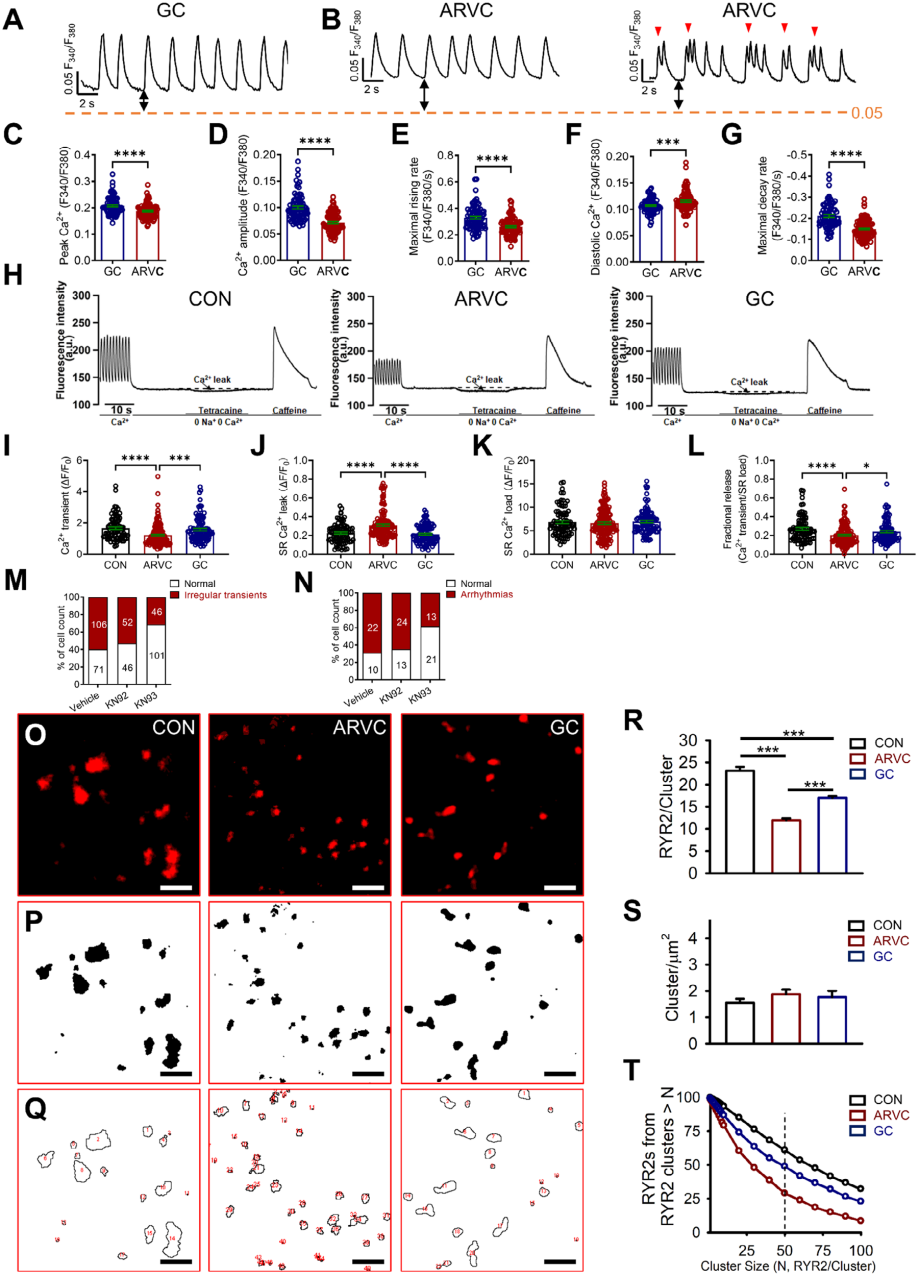
Decreased *RYR2* cluster size leads to abnormal SR Ca^2+^ release that is causally linked to the arrhythmic phenotypes in ARVC iPSC‐CMs. A,B) Representative Ca^2+^ transient tracings from GC and ARVC iPSC‐CMs. Red arrows indicate the irregular arrhythmia‐like Ca^2+^ transients. C–G) Bar graphs to compare key Ca^2+^ transient parameters between GC and ARVC iPSC‐CMs. *n* = 79–95 cells in 2 different iPSC lines. H) Representative traces of cytosolic Ca^2+^ fluorescence in control (CON), ARVC, and GC iPSC‐CMs in normal Tyrode's solution and exposed to 0 Na^+^, 0 Ca^2+^ solution containing 1 mm tetracaine and 10 mm caffeine. I–L) Bar graphs to compare the Ca^2+^ transient amplitude, SR Ca^2+^ leak, SR Ca^2+^ load, and fractional release between control, ARVC, and GC iPSC‐CMs. *n* = 87–148 cells in 2 different iPSC lines. M) Bar graph to compare the percentage of cells with irregular Ca^2+^ transients between ARVC iPSC‐CMs treated with DMSO (vehicle), KN92, and KN93. *n* = 98–177 cells in 2 different iPSC lines. N) Bar graph to compare the percentage of cells with arrhythmias between ARVC iPSC‐CMs treated with DMSO, KN92, and KN93. *n* = 32–37 cells in 2 different iPSC lines. O) Representative Tau‐STED imaging of *RYR2* (red) from control, ARVC, and GC iPSC‐CMs. P) Representative threshold binary images of (O). Q) Representative images showing *RYR2* clusters in (P) were identified and labeled. R) Bar graph to compare the mean cluster size between control, ARVC, and GC iPSC‐CMs. *n* = 1973–5343 clusters. Data were collected from 2 different iPSC lines. S) Bar graph to compare the mean cluster density between control, ARVC, and GC iPSC‐CMs. *n* = 10–23 cells in 2 different iPSC lines. T) The distribution of the number of *RYR2*s from clusters over a certain size across increasing cluster size. The vertical dashed line indicates the cluster size of 50. Scale bar, 500 nm. For all panels, data are represented as mean ± SEM. ^*^ *p* < 0.05; ^***^ *p* < 0.001; ^****^ *p* < 0.0001, unpaired two‐tailed Student's *t*‐test (C–G) or one‐way ANOVA followed by Tukey's HSD post‐hoc test (I–L, R, S).

As increased attention has been directed toward the role of *RYR2*‐mediated sarcoplasmic reticulum (SR) Ca^2+^ leak in arrhythmogenic cardiomyopathy,^[^ ^17^ ^]^ we next assessed intracellular Ca^2+^ handling in ARVC iPSC‐CMs by ratiometric Ca^2+^ imaging (**Figure** **3**A,B). ARVC iPSC‐CMs exhibited significantly reduced peak systolic Ca^2+^ and Ca^2+^ transient amplitude in ARVC iPSC‐CMs compared to GC iPSC‐CMs (Figure 3C,D), implicating systolic SR Ca^2+^ release was compromised. Maximal rising rate of Ca^2+^ transient was significantly decreased (Figure 3E), which could be resulted from reduced expression of *RYR2*. Diastolic Ca^2+^ was significantly elevated in ARVC iPSC‐CMs than in GC iPSC‐CMs (Figure 2F), suggesting *RYR2*‐mediated SR Ca^2+^ leak during diastole was enhanced. Activity of *SERCA2a*, as indicated by significantly decreased maximal decay rate (Figure 3G) and increased time constant of Ca^2+^ transient decay (Figure S9, Supporting Information), was also impaired in ARVC iPSC‐CMs as compared to GC iPSC‐CMs, which is consistent with reduced *SERCA2a* expression.

We next assessed *RYR2*‐mediated SR Ca^2+^ leak and SR Ca^2+^ load in control, ARVC, and GC iPSC‐CMs (Figure 3H). The transient amplitude was significantly reduced in ARVC iPSC‐CMs as compared to control and GC iPSC‐CMs (Figure 3I). *RYR2*‐mediated SR Ca^2+^ leak, as evidenced by the tetracaine‐induced Ca^2+^ shift, was significantly greater in ARVC iPSC‐CMs than in control and GC iPSC‐CMs (Figure 3J), which is in good agreement with aforementioned elevated diastolic Ca^2+^ in ARVC iPSC‐CMs. SR Ca^2+^ load, as indicated by the amplitude of caffeine‐induced Ca^2+^ transients, was smaller in ARVC iPSC‐CMs than in control and GC iPSC‐CMs but the difference was statistically insignificant (Figure 3K). Fractional SR Ca^2+^ release, defined as amplitude of the Ca^2+^ transient normalized to SR Ca^2+^ load, was significantly reduced in ARVC iPSC‐CMs (Figure 3L). Intriguingly, despite of being downregulated, the residual *RYR2*s were hyperphosphorylated at Ser2814 in ARVC iPSC‐CMs compared to GC iPSC‐CMs (Figure S7C,D, Supporting Information). Treatment of KN93, a specific *CaMKII* inhibitor, significantly suppressed the arrhythmic activities in ARVC iPSC‐CMs, when compared to ARVC myocytes treated with dimethyl sulfoxide (DMSO) (vehicle) or the inactive analogue KN92 (Figure 3M,N; Figure S10, Supporting Information). Collectively, these results suggest that abnormal SR Ca^2+^ release is causally linked to the arrhythmic phenotypes in ARVC iPSC‐CMs.

### 2.7 Decreased *RYR2* Cluster Size in ARVC iPSC‐CMs

*RYR2* clusters exhibit dynamic arrangement in cardiomyocytes and are susceptible to remodeling. In the present study, we employed Tau‐STED super‐resolution imaging to reveal morphological alteration in *RYR2* clusters in ARVC iPSC‐CMs at a resolution of ≈20 nm. Acquired raw Tau‐STED images (Figure 3O) were threshold (Figure 3P), *RYR2* clusters were automatically identified (Figure 3Q) and fitted to a 30 × 30 nm grid (corresponding to the structure of the *RYR2* protein) to assess *RYR2* cluster size.^[^ ^18^ ^]^ *RYR2* distribution pattern was compared in control, ARVC, and GC iPSC‐CMs. *RYR2* cluster size was significantly smaller in ARVC iPSC‐CMs (11.95 ± 0.42 *RYR2* per cluster) than in control (23.12 ± 0.84 *RYR2* per cluster, *p* < 0.001 vs ARVC) or GC iPSC‐CMs (17.00 ± 0.44 *RYR2* per cluster, *p* < 0.001 vs ARVC) (Figure 3R), with the cluster density being comparable among control (1.55 ± 0.16 cluster µm^−2^), ARVC (1.88 ± 0.17 cluster µm^−2^), and GC iPSC‐CMs (1.77 ± 0.24 cluster µm^−2^) (Figure 3S). Additionally, the reduction in mean cluster size observed in ARVC iPSC‐CMs is most likely due to the fragmentation of large clusters, as it was prominent that large clusters are relatively rare in ARVC iPSC‐CMs than in control and GC iPSC‐CMs, especially for the clusters containing 50 or more *RYR2*s (Figure S11, Supporting Information). As a result, ≈67% and 50% *RYR2* are from clusters containing over 50 *RYR2*s in control and GC iPSC‐CMs, the percentage was down to ≈25% in ARVC iPSC‐CMs (Figure 3T).

### 2.8 Subcellular Translocalization of *TMEM43* and Abnormal Nuclear Envelope Structure in P386S iPSC‐CMs

**Figure 4:**
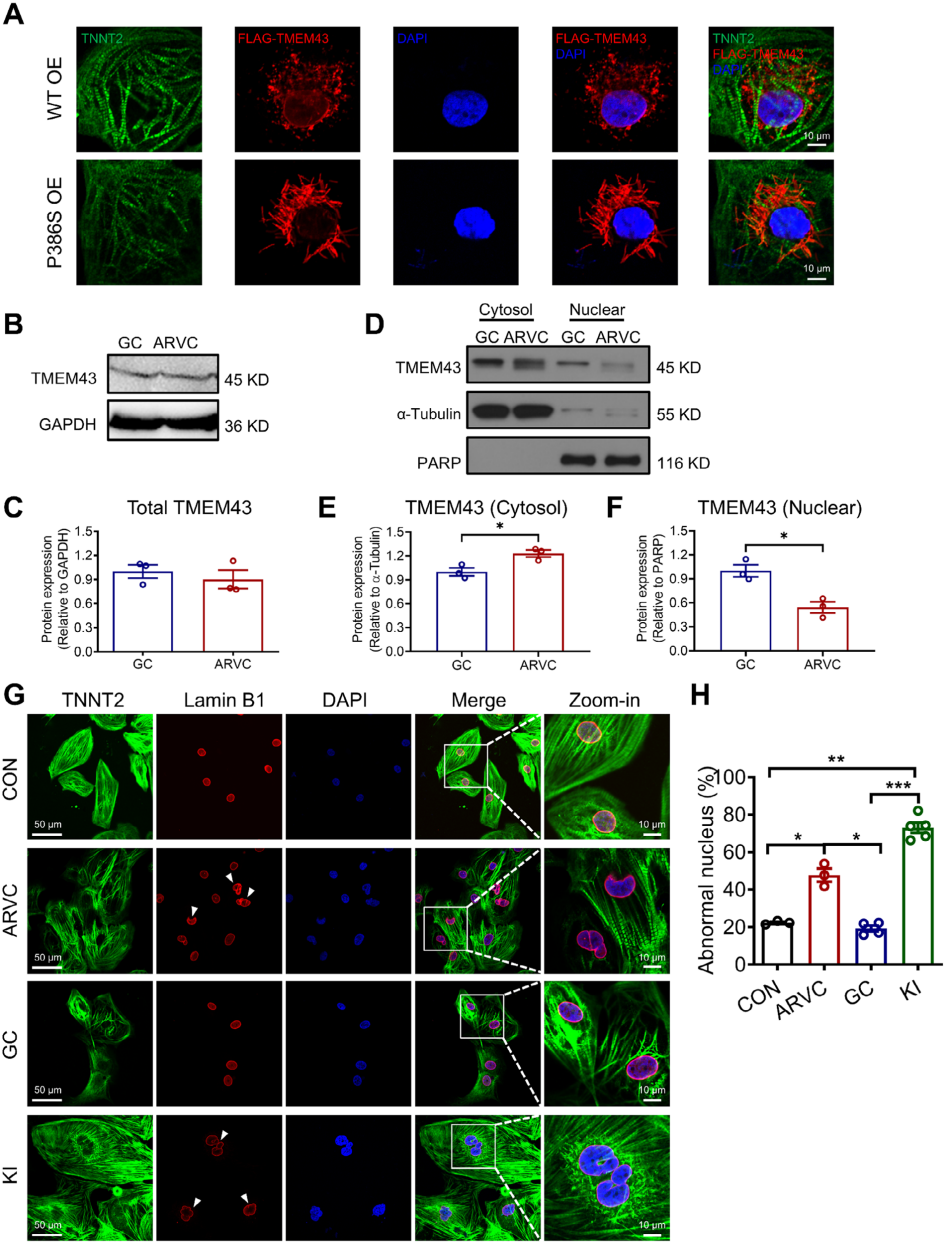
Subcellular translocalization of *TMEM43* and abnormal nuclear envelope structure in P386S iPSC‐CMs. A) Representative graphs of costaining by *TNNT2* (green) and FLAG *TMEM43* (red) in control iPSC‐CMs overexpressing lentiviral‐WT TMEM43‐GFP (WT OE) and lentiviral‐TMEM43‐P386S‐GFP (P386S OE). DAPI indicates nuclear staining (blue). *n* = 10–12 views in 3 independent experiments. B) Western blot analysis of total protein expression of *TMEM43* in GC and ARVC iPSC‐CMs. C) Bar graph to compare the total protein expression of *TMEM43* between GC and ARVC iPSC‐CMs. *n* = 3 batches of iPSC‐CMs from independent differentiations. D) Western blot analysis of protein expression of cytosol and nuclear *TMEM43* in GC and ARVC iPSC‐CMs. E,F) Bar graphs to compare the protein expression of cytosol and nuclear *TMEM43* between GC and ARVC iPSC‐CMs, respectively. *n* = 3 batches of iPSC‐CMs from independent differentiations. G) Representative graphs of costaining by *TNNT2* (green) and lamin B1 (red) in control, ARVC, and GC iPSC‐CMs. DAPI indicates nuclear staining (blue). White arrows indicate the abnormal nuclear envelope (NE) in ARVC iPSC‐CMs. H) Bar graph to compare the percentage of cells with abnormal nucleus between control, ARVC, GC, and KI iPSC‐CMs. *n* = 21–94 views in 3–5 independent experiments. Data were collected from 2 different iPSC lines. For all panels, data are represented as mean ± SEM. ^*^ *p* < 0.05; ^**^ *p* < 0.01; ^***^ *p* < 0.001, unpaired two‐tailed Student's *t*‐test (C, E, F) or one‐way ANOVA followed by Tukey's HSD post‐hoc test (H).

Mice model studies have suggested that *TMEM43* is localized predominantly to the nuclear envelope (NE) in cardiomyocytes.^[^ ^6^, ^19^ ^]^ We next infected control iPSC‐CMs with lentivirus containing either FLAG‐tagged WT *TMEM43* or TMEM43‐P386S to ascertain whether the mutation causes subcellular translocalization of *TMEM43* with confocal microscopy. In iPSC‐CMs overexpressing WT *TMEM43*, strong FLAG signals were detected at the NE, and punctate distribution of FLAG signals were also detected at the perinuclear region (**Figure** **4**A; Figure S12, Supporting Information). By contrast, FLAG signals localized at the NE were significantly reduced in iPSC‐CMs overexpressing mutant *TMEM43*, with perinuclear localization in a clustered manner (Figure 4A; Figure S12, Supporting Information). In addition, with the total protein expression levels being comparable between GC and ARVC iPSC‐CMs (Figure 4B,C), our results suggest a translocalization of *TMEM43* from the NE to the cytoplasm by the P386S mutation (Figure 4D–F).

*TMEM43* is tightly associated with the LINC complex,^[^ ^8^, ^20^ ^]^ which connects the nuclear lamina to the cytoskeleton, provides structural support to the nucleus, and plays an essential role in regulating gene expression and proteostasis.^[^ ^21^, ^22^, ^23^ ^]^ To investigate whether the translocalization of TMEM43‐P386S compromises the integrity of NE, lamin B1 was immunolabeled and the structure of NE was assessed with confocal microscopy (Figure 4G). As shown in Figure 4H, a significant proportion of ARVC and KI iPSC‐CMs displayed abnormal and wrinkled NE, with 47.8% and 73.0% of cells being affected, respectively; by contrast, only 22.3% of control iPSC‐CMs and 19.3% of GC iPSC‐CMs exhibited such abnormalities. Altogether, these results suggest that the P386S mutation causes distinct subcellular translocalization of *TMEM43* and abnormal NE structure in ARVC iPSC‐CMs.

### 2.9 Mislocalization of Lamin B2 Links to Decreased Chromatin Opening of Promoters around Downregulated Genes in ARVC iPSC‐CMs

**Figure 5:**
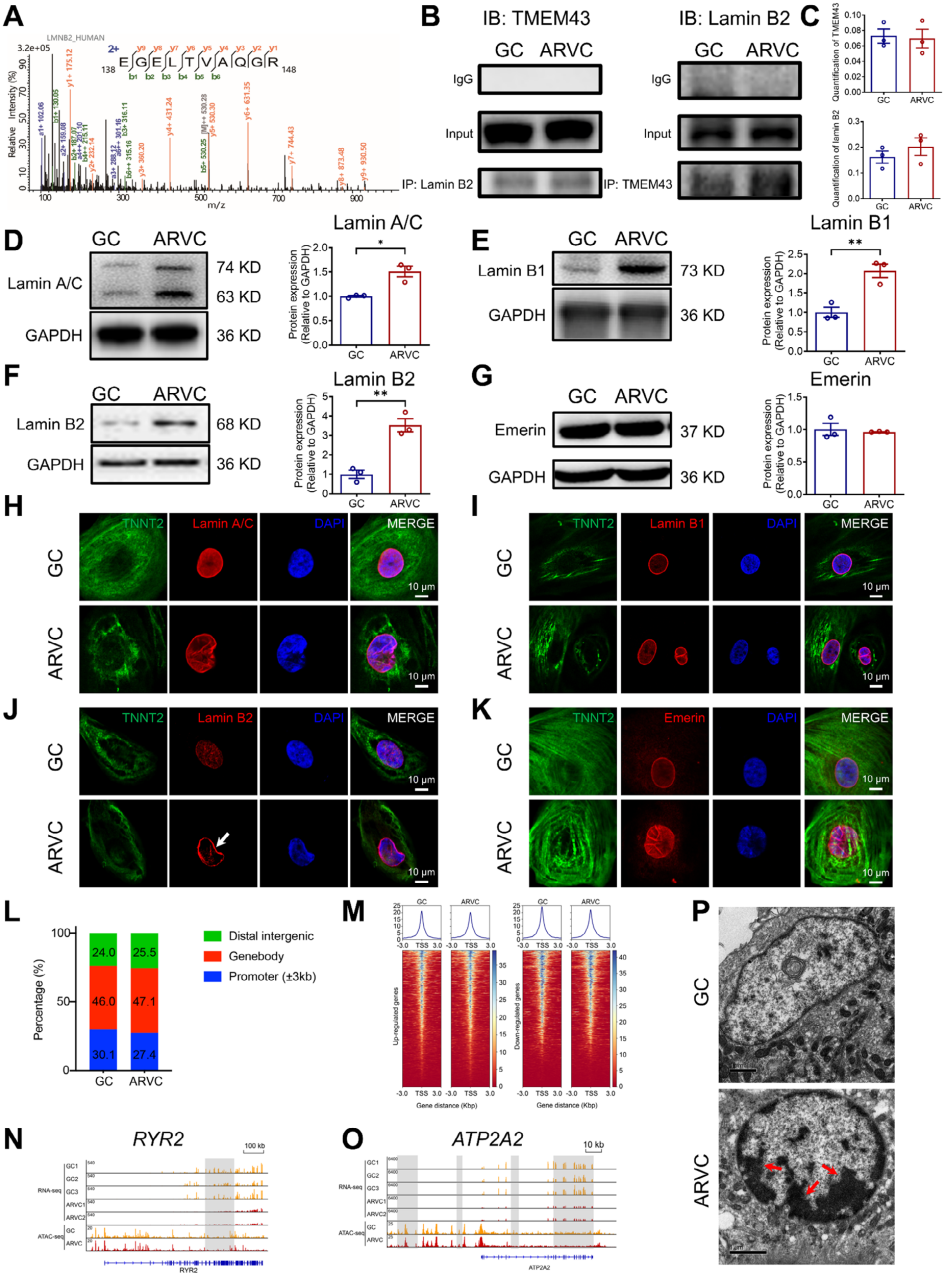
Mislocalization of lamin B2 links to decreased chromatin opening of promoters around downregulated genes in ARVC iPSC‐CMs. A) Mass spectrum of lamin B2 peptides. B) Co‐immunoprecipitation (co‐IP) studies to detect interaction between *TMEM43* and lamin B2 in GC and ARVC iPSC‐CMs using *TMEM43* antibody, lamin B2 antibody, or IgG (negative control antibody). C) Bar graphs to compare the protein–protein interaction of *TMEM43* and lamin B2 between GC and ARVC iPSC‐CMs. *n* = 3 batches of iPSC‐CMs from independent differentiations. D–G) Left panel, Western blot analysis of lamin A/C, lamin B1, lamin B2, and emerin in GC and ARVC iPSC‐CMs. Right panel, bar graphs to compare the protein expression of lamin A/C, lamin B1, lamin B2, and emerin between GC and ARVC iPSC‐CMs. *n* = 3 batches of iPSC‐CMs from independent differentiations. H–K) Representative graphs of costaining by *TNNT2* (green) and different LINC proteins (lamin A/C, lamin B1, lamin B2, and emerin) (red) in GC and ARVC iPSC‐CMs. DAPI indicates nuclear staining (blue). The white arrow indicates the mislocalization of lamin B2 in ARVC iPSC‐CMs. *n* = 24–28 views in 3 independent experiments. Data were collected from 2 different iPSC lines. L) ATAC‐Seq peak distribution on the genome. Promoter was defined as 3 kb upstream and downstream region of gene transcription start site (TSS). M) Normalized ATAC‐Seq signal intensity around promoter of up‐ and downregulated genes in GC and ARVC iPSC‐CMs. N,O) Genome browser views of ATAC‐Seq and RNA‐Seq data on *RYR2* and *ATP2A2* loci in ARVC iPSC‐CMs as compared to GC iPSC‐CMs. P) Representative graphs of electron microscope in GC and ARVC iPSC‐CMs. Red arrows indicate peripheral condensation of chromatin in ARVC iPSC‐CMs. For all panels, data are represented as mean ± SEM. ^**^ *p* < 0.01, unpaired two‐tailed Student's *t*‐test (C–G).

To understand the mechanism of how TMEM43‐P386S confers the structurally abnormal NE, we next analyzed the *TMEM43* interactome via mass spectrometry. Immunoprecipitation assay was performed in control, ARVC, and GC iPSC‐CMs using a *TMEM43* antibody. Of the *TMEM43*‐associated proteins identified in control, ARVC, and GC iPSC‐CMs, 4 were desmosome proteins (plakophilin 2 (*PKP2*), junction plakoglobin (*JUP*)) or lamin proteins (lamin A/C, lamin B1) that were previously reported.^[^ ^24^, ^25^, ^26^ ^]^ LINC complex, consisting of nesprins, SUNs, emerin, *TMEM43*, and lamins, provides structural support to the nucleus and physically couples the nucleoskeleton with the cytoskeleton. Interestingly, we also identified lamin B2, one of the major components in LINC complex, as a *TMEM43*‐binding protein with multiple assigned peptides (**Figure** **5**A; Table S3, Supporting Information). We next sought to validate the mass spectrometry findings implicating lamin B2 as a novel *TMEM43* interactor. Reciprocal co‐immunoprecipitation (co‐IP) experiments using either *TMEM43* or lamin B2 antibody confirmed the interaction between *TMEM43* and lamin B2 in both GC and ARVC iPSC‐CMs (Figure 5B). Furthermore, TMEM43‐P386S does not influence the interaction between *TMEM43* and lamin B2 proteins (Figure 5C).

To test if TMEM43‐P386S could affect expression and nuclear localization of LINC‐complex‐associated proteins, Western blot analysis and immunofluorescent staining were performed utilizing a panel of different antibodies in isogenic iPSC‐CMs. The expression levels of lamin A/C, lamin B1, and lamin B2 were all significantly increased in ARVC iPSC‐CMs compared to GC iPSC‐CMs, with emerin being unaltered (Figure 5D–G). Surprisingly, subcellular localization of lamin B2, but not lamin A/C, lamin B1, or emerin, was strikingly altered in ARVC iPSC‐CMs as compared to GC iPSC‐CMs, manifesting predominant localization at the NE with diminished nucleocytoplasmic localization (Figure 5H–K; Figure S13, Supporting Information).

Lamins regulate chromatin organization and gene expression and influence cell signaling.^[^ ^8^ ^]^ To investigate whether the disrupted localization of lamin B2 could affect chromatin accessibility and openness, we next performed assay for transposase‐accessible chromatin with high throughput sequencing (ATAC‐Seq) to profile the open chromatin in ARVC iPSC‐CMs. We observed a genome‐wide redistribution and a concomitant decrease of chromatin opening in gene promoters in ARVC iPSC‐CMs (Figure 5L). Further, combined analysis with RNA‐Seq data, heatmap of ATAC‐Seq signals around the promoters of DEGs showed that the promoters of downregulated genes were associated with decreased open chromatin in ARVC iPSC‐CMs in comparison to GC iPSC‐CMs. Promoters of upregulated genes had no significant changes with open chromatin (Figure 5M). Representative gene locus showed consistent results (Figure 5N,O). Downregulated genes in ARVC iPSC‐CMs, including *RYR2* and *ATP2A2* associated with decreased open chromatin (Figure 5N,O). Moreover, transmission electron microscopy revealed that a part of chromatin was migrated to the cell edge, exhibiting peripheral condensation of chromatin in ARVC iPSC‐CMs, when compared to GC iPSC‐CMs (Figure 5P). Collectively, these results suggest that TMEM43‐P386S causes mislocalization of lamin B2, which confers the abnormal NE structure and decreased chromatin opening of promoters around downregulated genes, including *RYR2*.

### 2.10 Lamin B2 Depletion Recapitulates Arrhythmic Phenotypes of ARVC iPSC‐CMs

Moving forward, using control iPSCs as a template, we successfully generated lamin B2 knockout (KO) iPSC lines by CRISPR/Cas9‐mediated genome editing (Figure S14, Supporting Information). Western blot analysis indicated a translocation of *TMEM43* protein from nucleus to cytoplasm in lamin B2 KO iPSC‐CMs compared to their control counterparts (Figure S15A–C, Supporting Information). RNA‐Seq analysis demonstrated a distinct transcriptome profile in lamin B2 KO iPSC‐CMs, which was similar with that in ARVC iPSC‐CMs (Figure S15D–H, Supporting Information). Importantly, lamin B2 KO iPSC‐CMs exhibited arrhythmic phenotypes and abnormal action potential profile in comparison to control iPSC‐CMs (Figure S15I,J and Table S4, Supporting Information). Ca^2+^ imaging validated the arrhythmic phenotypes and revealed an irregular Ca^2+^ handling profile (Figure S15K–Q, Supporting Information). Such action potential and Ca^2+^ transient recordings resembled those from ARVC iPSC‐CMs. Taken together, these results suggest that lamin B2 depletion recapitulates arrhythmic phenotypes of ARVC iPSC‐CMs.

### 2.11 Flecainide Exerts an Antiarrhythmic Effect on ARVC iPSC‐CMs

**Figure 6:**
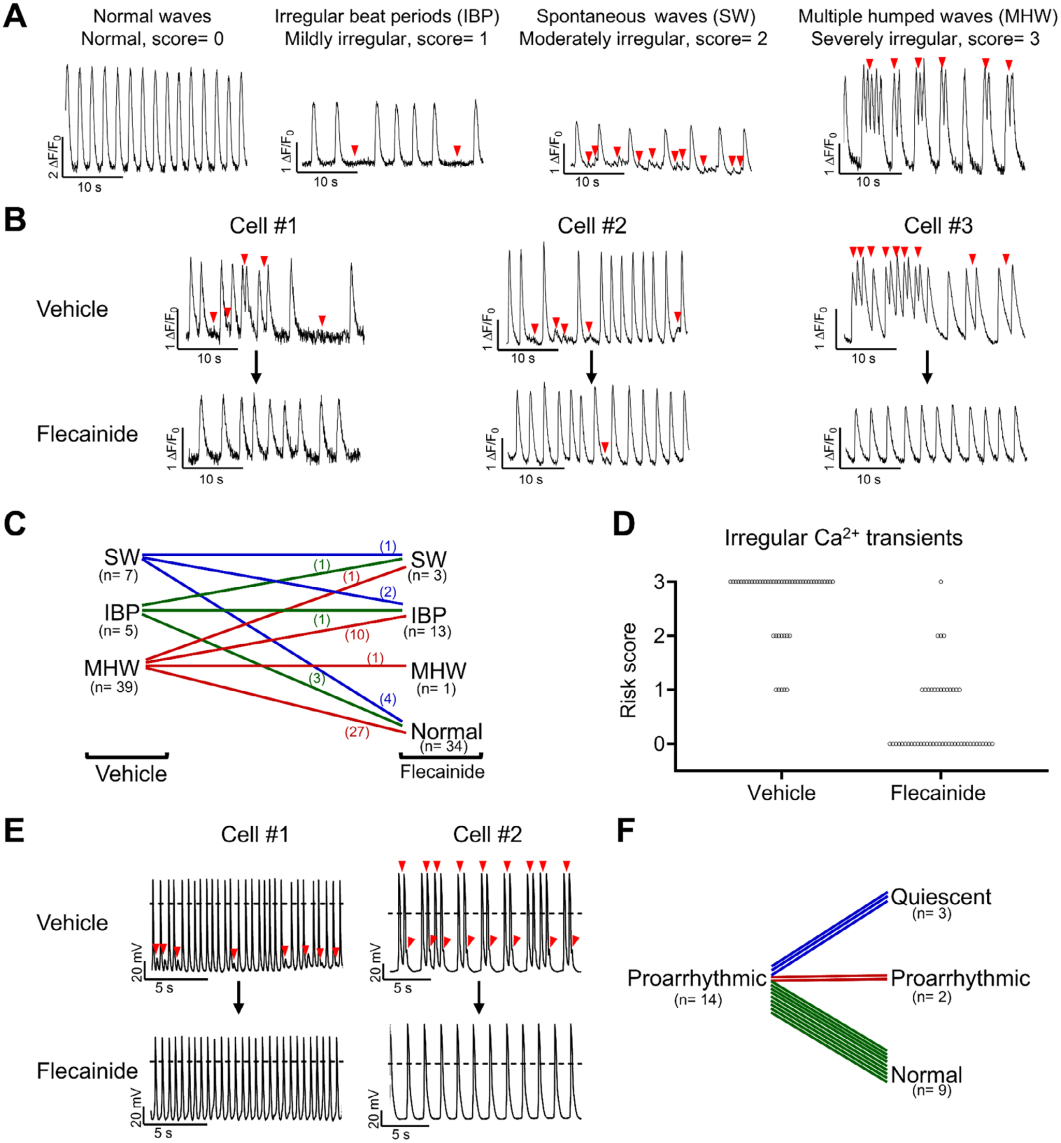
Flecainide exerts an antiarrhythmic effect on ARVC iPSC‐CMs. A) Ca^2+^ transient abnormality types in ARVC iPSC‐CMs. Normal waves: Normal; irregular beat periods: IBP; spontaneous waves: SW; multiple humped waves: MHW. Risk scores for Normal (regular), IBP (mildly irregular), SW (moderately irregular), and MHW (severely irregular) are 0, 1, 2, and 3, respectively. Red arrows indicate irregular Ca^2+^ transients. B) Representative Ca^2+^ transient tracings from ARVC iPSC‐CMs before and after flecainide treatment. Cell #1, Cell #2, and Cell #3 denote the 3 representative cells for flecainide testing. Red arrows indicate irregular Ca^2+^ transients. C) Classification of irregular Ca^2+^ transient risk for vehicle (DMSO) and flecainide treatment groups. D) Risk score of irregular Ca^2+^ transients for vehicle (DMSO) and flecainide treatment groups. *n* = 51 cells in 2 different iPSC lines. E) Representative action potential tracings from ARVC iPSC‐CMs before and after flecainide treatment. Cell #1 and Cell #2 denote the 2 representative cells for flecainide testing. F) Classification of arrhythmia risk for vehicle (DMSO) and flecainide treatment groups. Red arrows indicate arrhythmias.

The class Ic/IVb antiarrhythmic drug flecainide has emerged as an effective alternative in patients with catecholaminergic polymorphic ventricular tachycardia, a genetic arrhythmic disorder of *RYR2* hyperactivity.^[^ ^27^, ^28^, ^29^ ^]^ The hyperactive *RYR2* phenotype in ARVC iPSC‐CMs prompted us to investigate whether flecainide could suppress the impact of TMEM43‐P386S in ARVC iPSC‐CMs. Fluo‐4 Ca^2+^ imaging assay was performed on the same myocytes before and after acute treatment of flecainide (10 µm), and irregular Ca^2+^ transients were classified on an ordinal scale from 0 to 4 (0: normal; 1: irregular beat periods, mildly irregular; 2: spontaneous waves, moderately irregular; 4: multiple humped waves, severely irregular) (**Figure** **6**A). 76% (39 of 51) of the recorded ARVC iPSC‐CMs exhibited severely irregular Ca^2+^ transients at baseline (Figure 6B–D). By contrast, flecainide treatment significantly ameliorated the irregular Ca^2+^ transient phenotype in ARVC iPSC‐CMs, with 27 and 10 myocytes demonstrating normal and mildly irregular Ca^2+^ transients, respectively (Figure 6B–D). In addition, the proarrhythmic activities in ARVC iPSC‐CMs were greatly attenuated when treated with flecainide as evidenced by patch clamp recordings (Figure 6E). Flecainide completely abolished all DAD arrhythmias in 64% (9 of 14) of the tested ARVC myocytes (Figure 6F). Altogether, these results indicate that flecainide exerts an antiarrhythmic effect on ARVC iPSC‐CMs.

### 2.12 Flecainide Prevents Cardiac Arrhythmias in TMEM43‐P386S KI Mice under Sympathetic Stimulation

**Figure 7:**
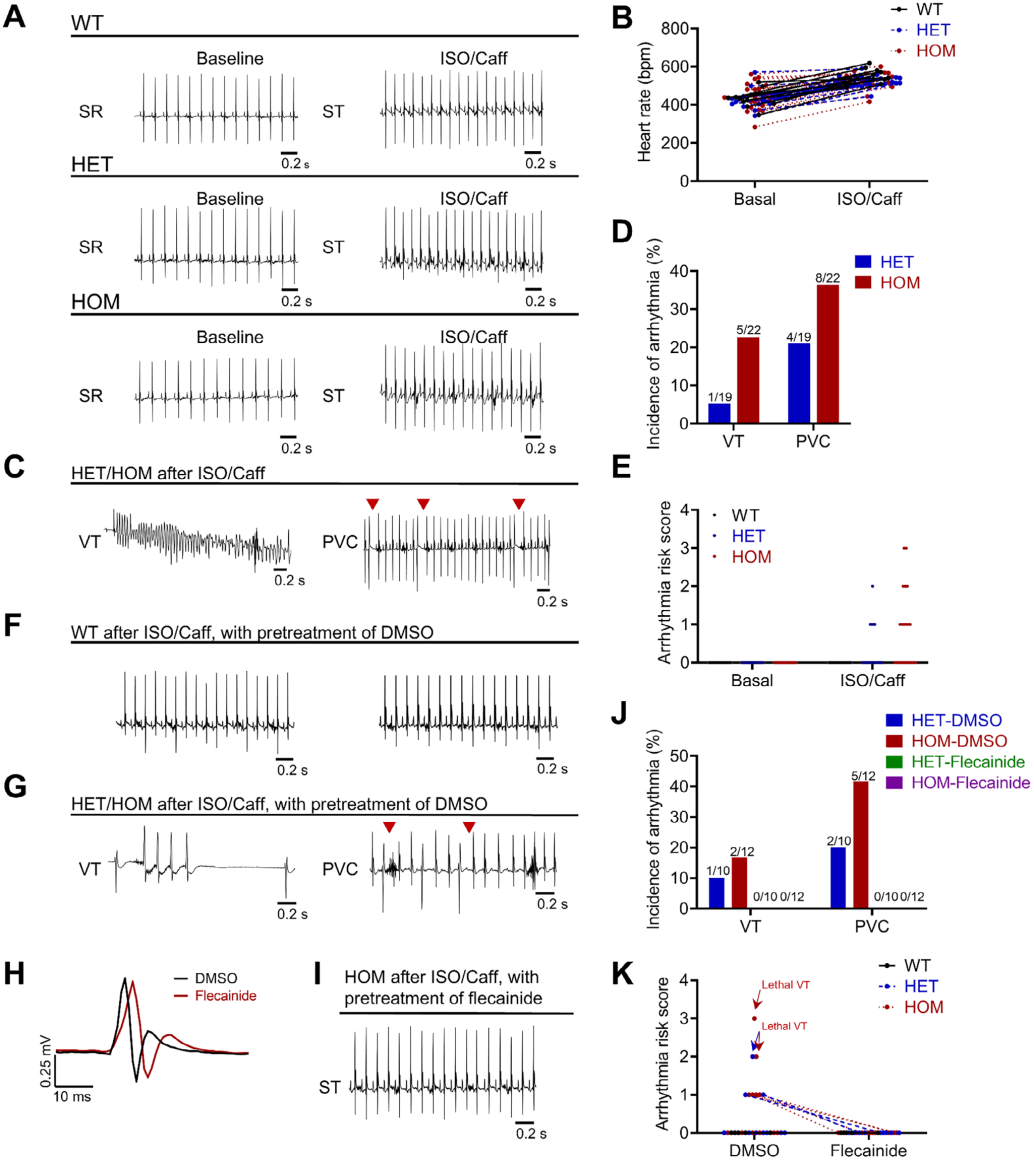
Pretreatment of flecainide prevents cardiac arrhythmias in the TMEM43‐P386S KI mouse model after sympathetic stimulation. A) Representative surface ECG recordings of wild type (WT), heterozygous (HET), and homozygous (HOM) KI mice at baseline and during stimulation by ISO (2 mg kg^−1^ body weight) and caffeine (Caff) (120 mg kg^−1^ body weight). SR, sinus rhythm; ST, sinus tachycardia. B) Quantification of heart rate of WT, HET, and HOM KI mice at baseline and during sympathetic stimulation. *n* = 11 in WT mice, *n* = 19 in HET mice, *n* = 22 in HOM mice. C) Arrhythmias recorded in KI mice after sympathetic stimulation. PVC, premature ventricular contraction; VT, ventricular tachycardia. Red arrows indicate the PVCs. D) Incidence of different types of arrhythmia calculated from HET and HOM KI mice in response to ISO/Caff. E) Arrhythmia risk score of different types of mice. Arrhythmia risk score was classified as 0 = no ectopy, 1 = PVC, 2 = VT. F) Representative surface ECG recordings of WT mice under ISO/Caff stimulation with pretreatment of DMSO. G) Arrhythmias recorded in KI mice under ISO/Caff stimulation with pretreatment of DMSO. Red arrows indicate the PVCs. H) Representative ECG traces illustrating QRS morphology from the same HOM KI mouse treated with DMSO and flecainide. Waveforms are aligned to the initial moment of depolarization. I) Representative surface ECG recordings of HOM KI mice under ISO/Caff stimulation with pretreatment of flecainide. J) Incidence of different types of arrhythmia calculated from HET and HOM KI mice in response to ISO/Caff with pretreatment of DMSO or flecainide. K) Arrhythmia risk score of different types of mice under ISO/Caff stimulation with pretreatment of DMSO or flecainide. *n* = 5 in WT mice, *n* = 10 in HET mice, *n* = 12 in HOM mice.

To investigate the in vivo effects of TMEM43‐P386S on cardiac electrophysiology, we therefore generated a KI mouse model harboring this mutation using the CRISPR/Cas9 system (Figure S16A, Supporting Information). The presence of the TMEM43‐P386S mutation in KI mice was confirmed by polymerase chain reaction (PCR) and sequencing analysis (Figure S16B, Supporting Information). Next, we conducted electrophysiological studies on WT and KI mice at 4 months of age. At baseline, the ECG profiles were comparable among WT, heterozygous, and homozygous mice with no arrhythmic events (**Figure** **7**A). However, distinct differences emerged between WT and KI groups when the mice were subjected to stimulation by isoproterenol and caffeine (ISO/Caff). While WT mice only exhibited a significantly accelerated heart rate without experiencing arrhythmias (Figure 7B), a portion of KI mice developed arrhythmias under the stimulation conditions (Figure 7C).

In the heterozygous group, 5 out of 19 mice displayed arrhythmias under ISO/Caff stimulation. Specifically, four mice developed premature ventricular contraction (PVC) and one mouse developed VT (Figure 7D). Moreover, the homozygous mice exhibited the most severe arrhythmic phenotype, with 11 out of 22 mice displaying stress‐induced arrhythmias. Among these mice, six displayed PVC, three developed lethal VT, and two had both PVC and VT (Figure 7D). Compared to WT mice, the KI mice showed a marked increase in the arrhythmia risk score under the stimulation condition (Figure 7E).

Next, the in vivo antiarrhythmic effect of flecainide was assessed during ISO/Caff stimulation. The WT or KI mice were pretreated with 5% DMSO (as vehicle control) or 20 mg kg^−1^ flecainide. In vehicle groups, stress‐induced arrhythmias were only observed in KI mice, but not in WT mice (Figure 7F,G). In flecainide groups, prolonged QRS complex was observed in both WT and KI mice, which was consistent with previous studies (Figure 7H).^[^ ^27^, ^30^ ^]^ Notably, none of the KI mice pretreated with flecainide displayed ventricular arrhythmias (Figure 7I,J). Pretreatment of flecainide greatly reduced the arrhythmia risk score in KI mice under ISO/Caff stimulation (Figure 7K). These results demonstrate that flecainide can prevent stress‐induced cardiac arrhythmias in TMEM43‐P386S KI mice.

### 2.13 Flecainide Suppresses Spontaneous SR Ca^2+^ Release in TMEM43‐P386S KI Mouse Myocytes

**Figure 8:**
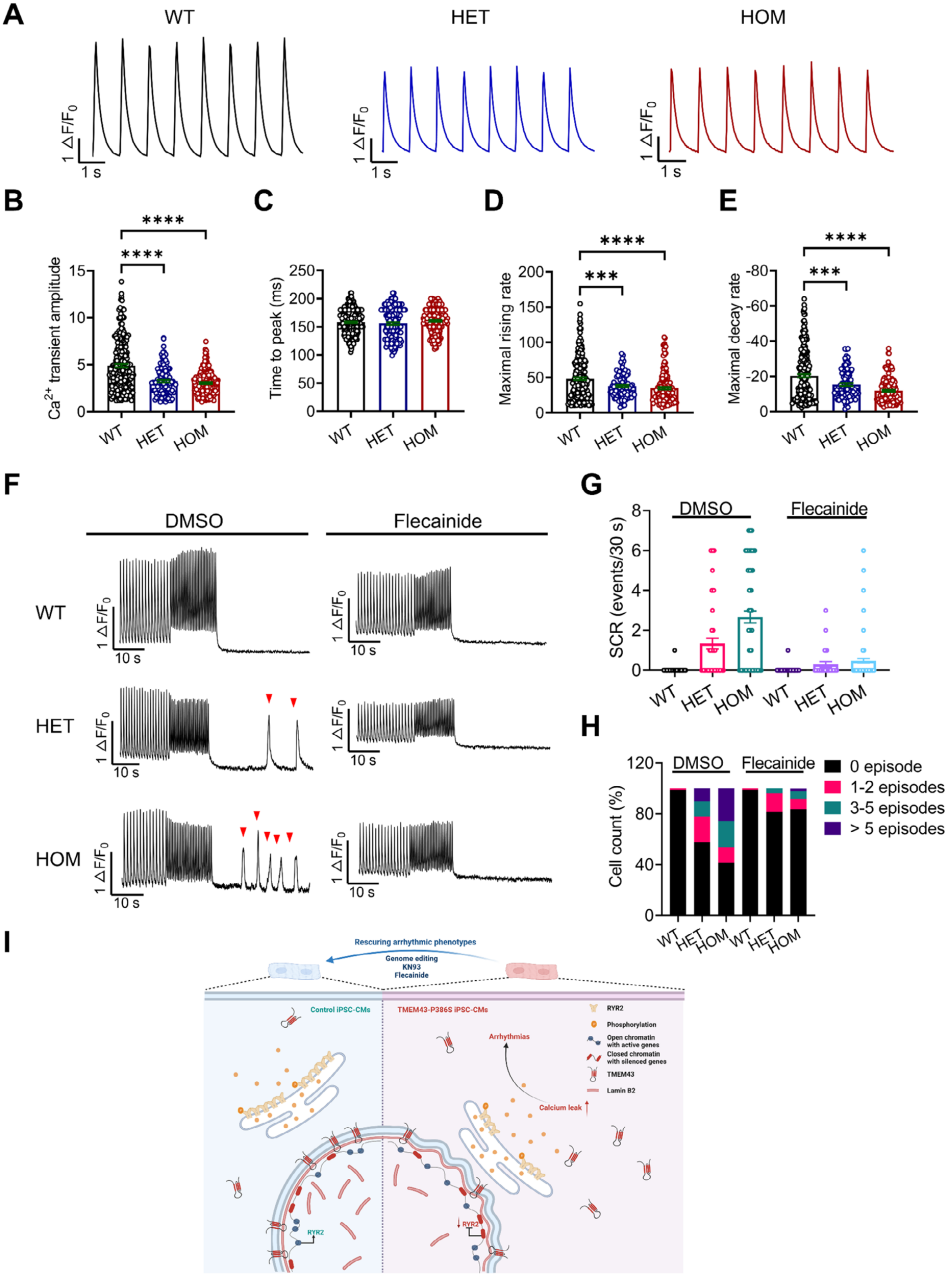
Flecainide suppresses spontaneous SR Ca^2+^ release in TMEM43‐P386S KI mouse cardiomyocytes. A) Representative Ca^2+^ transient tracings recorded by Fluo‐4 Ca^2+^ imaging of ventricular myocytes isolated from 4 months old WT and KI hearts. B–E) Bar graphs to compare Ca^2+^ transient amplitude, time to peak, maximal rising rate, and maximal decay rate between WT and KI cardiomyocytes. *n* = 99–237 cells per group from 3–5 hearts per genotype. F) Representative Ca^2+^ transient tracings (1–2 Hz field stimulation to steady then pause) recorded by Fluo‐4 Ca^2+^ imaging of ventricular myocytes isolated from 4 months old WT and KI hearts with pretreatment of DMSO or 5 µm flecainide. Red arrows indicate the spontaneous Ca^2+^ release (SCR) from SR. G) Frequency of SCRs during 30 s period after cessation of stimulation in the absence and presence of 5 µm flecainide. H) Incidence of cardiomyocytes (WT and KI) with 0 SCR episode, 1–2 episodes, 3–5 episodes, and more than 5 episodes in the absence and presence of 5 µm flecainide. *n* = 27–134 cells per group from 4 hearts per genotype. I) Proposed work model. WT *TMEM43* locates at the NE and cytoplasmic region in iPSC‐CMs, whereas P386S mutant *TMEM43* is translocated from the NE to the cytoplasm. *TMEM43* interacts with the NE protein lamin B2, and TMEM43‐P386S leads to lamin B2 mislocalization and abnormal NE structure in ARVC iPSC‐CMs, resulting in decreased chromatin opening of promoters around downregulated genes, including *RYR2*. *RYR2*s are downregulated, hyperphosphorylated, and grouped into smaller clusters in ARVC iPSC‐CMs, contributing to enhanced *RYR2*‐mediated SR Ca^2+^ leak. The resultant augmentation of SR Ca^2+^ release causes the arrhythmias, which can be alleviated by antiarrhythmic flecainide or *CaMKII* inhibitor KN93 for stabilization of *RYR2*. For all panels, data are represented as mean ± SEM., ^***^ *p* < 0.001; ^****^ *p* < 0.0001, one‐way ANOVA with Dunnett's multiple comparison test versus control (WT cardiomyocytes) (B–E).

Heart tissues were collected from both WT and KI mice for quantitative real‐time PCR (qPCR) and Western blot analyses. The mRNA levels of *RYR2* and *ATP2A2* in homozygous KI group were significantly lower than those in WT group (Figure S17A, Supporting Information). The protein levels of total *RYR2* and *SERCA2a* in heterozygous and homozygous KI groups were significantly lower as compared to WT group, while the phosphorylation levels of *RYR2* at Ser2814 were significantly higher in KI group (Figure S17B,C, Supporting Information). These data were consistent with our observations in the iPSC‐CM model. Immunofluorescence analysis revealed significantly reduced expression of *RYR2* in enzymatically isolated ventricular myocytes from homozygous KI group, when compared to WT group (Figure S17D,E, Supporting Information). To further investigate the underlying mechanisms, we next assessed Ca^2+^ handling characteristics in enzymatically isolated ventricular myocytes from mouse. As shown in **Figure** **8**A–E, KI mice exhibited significantly reduced Ca^2+^ transient amplitude, as well as diminished Ca^2+^ rising rate and decay rate in comparison to the WT group, with time to peak being similar among all groups. Remarkably, akin to what was observed in iPSC‐CMs, ventricular myocytes from homozygous KI mice exhibited significantly increased incidence of spontaneous Ca^2+^ release (SCR) following pacing training compared to WT mice, with heterozygous mice being intermediate (Figure 8F–H). Notably, treatment of flecainide greatly alleviated SCR observed in KI mice, not only the percentage of SCR‐positive cells was significantly reduced in the presence of flecainide, the incidence of SCR events was suppressed by flecainide in both heterozygous and homozygous myocytes as well (Figure 8F–H). To better elucidate the mechanism underlying the antiarrhythmic effects of flecainide in our mouse model, we assessed Ca^2+^ spark frequency in ventricular myocytes and assessed the impact of flecainide on Ca^2+^ sparks. Our findings revealed a significant increase in Ca^2+^ spark frequency in homozygous myocytes as compared to WT, which can be effectively suppressed by flecainide treatment (Figure S18, Supporting Information).

## 3 Discussion

Here, we employed the human iPSC‐CM model to uncover cellular phenotypes and elucidate molecular mechanisms underlying increased cardiac arrhythmogenesis in *TMEM43*‐related ARVC. We demonstrated that a novel *TMEM43* mutation (TMEM43‐P386S) causes abnormal SR Ca^2+^ release that led to arrhythmic phenotypes in ARVC‐patient‐specific iPSC‐CMs. We identified that *TMEM43* interacts with lamin B2, and TMEM43‐P386S causes mislocalization of lamin B2 and abnormality in NE structure in ARVC iPSC‐CMs, resulting in decreased chromatin opening of promoters around downregulated genes, including *RYR2*. We further showed that *RYR2*s are downregulated, hyperphosphorylated, and grouped into smaller clusters in ARVC iPSC‐CMs, as revealed by tau‐STED super‐resolution imaging, contributing to enhanced *RYR2*‐mediated SR Ca^2+^ leak. Conversely, *RYR2* stabilization by antiarrhythmic flecainide or *CaMKII* inhibitor KN93 effectively rescued the arrhythmic phenotypes of ARVC iPSC‐CMs.

Mutations in the *TMEM43* gene were described to cause ARVC5. TMEM43‐S358L was originally reported as the disease‐causing mutation in 15 extended families in the island of Newfoundland in Canada, which was associated with high penetrance and high risk of SCD and heart failure, with poor R‐wave progression and frequent LV dilatation.^[^ ^9^, ^31^ ^]^ Genetic mice models to study TMEM43‐S358L were recently generated, but their phenotypes were highly inconsistent.^[^ ^6^, ^19^, ^32^ ^]^ So far, the molecular links between *TMEM43* mutations and increased cardiac arrhythmogenesis remain largely elusive.

We provided robust evidence showing in vitro iPSC‐CM model can recapitulate the arrhythmic phenotypes of *TMEM43*‐related ARVC. Patient‐specific iPSC‐CMs carrying TMEM43‐P386S exhibited a series of baseline electrophysiological abnormalities at the single‐cell and multicellular levels, including DAD and PCF arrhythmias, beating interval variation, slower depolarization, and conduction defect. In line with previous clinical studies,^[^ ^15^, ^16^ ^]^ ARVC iPSC‐CMs were more susceptible to β‐adrenergic challenge and application of ISO in these cells caused more severe arrhythmias. Successful correction of TMEM43‐P386S at the iPSC level by CRISPR/Cas9‐mediated genome editing technique allowed for generation and characterization of isogenic control iPSC‐CMs to evaluate the pathogenicity of the P386S mutation. The resultant GC iPSC‐CMs exhibited significant improvement of arrhythmic phenotypes as compared to those observed in ARVC iPSC‐CMs, indicating that TMEM43‐P386S is a pathogenic mutation to cause arrhythmias in ARVC.

Unbiased RNA‐Seq analysis by comparing isogenic iPSC‐CMs indicated that Ca^2+^ signaling pathway is significantly changed in ARVC iPSC‐CMs. Western blot analysis revealed that protein expression levels of total *SERCA2a* and *RYR2* were significantly reduced in ARVC iPSC‐CMs. These observations were in line with previous evidence that altered Ca^2+^ handling gene and protein expression occurred in ARVC.^[^ ^33^, ^34^, ^35^, ^36^ ^]^ Functionally, ARVC iPSC‐CMs exhibited a markedly enhanced *RYR2*‐mediated SR Ca^2+^ leak, elevated diastolic Ca^2+^, irregular Ca^2+^ transients and arrhythmias. Defects in Ca^2+^ handling are responsible for dysregulated Ca^2+^ homeostasis and cardiac arrhythmias, and occurrence of DADs is associated with intracellular Ca^2+^ overload.^[^ ^37^ ^]^ We thus reasoned that Ca^2+^ handling abnormality engendered by TMEM43‐P386S is responsible for the arrhythmic phenotypes in ARVC.

A striking finding here was that the decreased *RYR2* expression was companied with a reduction in *RYR2* cluster size in ARVC iPSC‐CMs, whereas the density of *RYR2* clusters was only insignificantly reduced. Interestingly, the *RYR2*s were hyperphosphorylated by *CaMKII* at S2814 in ARVC iPSC‐CMs. A recent study demonstrated that prolonged *CaMKII* phosphorylation could be the driving force of the fragmentation of *RYR2* clusters.^[^ ^38^ ^]^ Therefore, on top of the reduced *RYR2* expression, the hyperphosphorylation of *RYR2*s by *CaMKII* may further exacerbate the fragmentation of *RYR2* clusters in our case.

The findings underscore the critical role of aberrant Ca^2^⁺ handling in the genesis of ventricular arrhythmias within our model. Despite our Ca^2^⁺ transient data strongly support the notion that dysregulated Ca^2^⁺ release through *RYR2* channels is a key factor in the development of arrhythmic events, the intricate balance within the intracellular Ca^2^⁺ homeostatic machinery extends beyond *RYR2* activity alone. The SR has a comprehensive effect on cytoplasmic Ca^2^⁺ levels primarily through the orchestrated actions of *RYR2*, *SERCA2a*, and the activity of their regulatory proteins or posttranslational modifications. While *SERCA2a* pumps and *PLN*, which inhibits *SERCA2a* activity and whose dephosphorylation enhances *SERCA2a* function, are essential for the efficient reuptake of cytoplasmic Ca^2^⁺ back into the SR, their role in modulating arrhythmogenic potential were not adequately addressed in our study. Moreover, the interaction between *CaMKII* and *RYR2* is pivotal in the context of ventricular arrhythmias and heart failure. *CaMKII*‐mediated phosphorylation of *RYR2* can lead to increased channel sensitivity to intracellular Ca^2^⁺, potentially predisposing the heart to arrhythmic events.^[^ ^39^ ^]^ The use of KN93, a selective inhibitor of *CaMKII*, has been shown to reduce arrhythmic incidence, indicating the therapeutic potential of targeting this pathway. Recent findings indicate that enhanced phosphorylation at Ser2814 leads to a more dispersed arrangement of *RYR2* channels.^[^ ^40^ ^]^ Moore and co‐workers’ research elucidates that the clustering of *RYR2*, the Ca^2^⁺ release channels, is influenced by their phosphorylation status, revealing a new mechanism by which phosphorylation regulates *RYR2* function. This discovery underscores the importance of *RYR2* phosphorylation levels on the organization of their clusters, potentially representing a novel pathway for controlling intracellular Ca^2^⁺ release in cardiomyocytes, which in turn modulates cardiac contraction. Through this mechanism, *RYR2* phosphorylation not only alters the activity of individual channels but also influences the overall cardiac physiology by changing their spatial organization. Such shifts in *RYR2* distribution due to phosphorylation alterations may contribute to increased Ca^2^⁺ leakage from the SR. Recent work in rabbit hearts expressing ≈50% of *RYR2* without any accompanying changes in cluster size or phosphorylation levels showed little change in function at the cellular and organ levels.^[^ ^41^ ^]^ Integrating the insights from this study with ours, it may become evident that the size of *RYR2* clusters could be a pivotal factor in determining the phenotypic differences observed in these models, and the efficacy of *RYR2*‐mediated Ca^2^⁺ release could be more dependent on phosphorylation levels and cluster size of *RYR2*s, rather than the total number of channels. Therefore, elucidating the *CaMKII*–*RYR2* interaction may provide insights into novel therapeutic strategies aimed at mitigating ventricular arrhythmias associated with heart failure.

The *RYR2*s in a cluster are expected to be functionally coupled, so that the opening or closing of one channel enhances the rate constant for opening or closing of neighboring channels in the same cluster.^[^ ^42^, ^43^ ^]^ This cooperative activity is thought to be important in determining the duration of the release event and may be altered by phosphorylation of *RYR2*.^[^ ^42^, ^43^, ^44^, ^45^ ^]^ Theoretically, cooperative gating of *RYR2* favors a steeper Ca^2+^ dependence for *RYR2* activation comparing with the activation of single *RYR2*s, thus *RYR2*s in a larger cluster are more likely to be stable at diastolic Ca^2+^ to prevent spontaneous SR Ca^2+^ leak during diastolic refilling of SR.^[^ ^46^ ^]^ Indeed, no published gating schemes of individual *RYR2* can simultaneously explain the systolic and diastolic features of cardiac excitation–contraction coupling, whereas introducing cooperative gating among arrayed *RYR2*s in a cluster effectively resolved the problem.^[^ ^47^ ^]^

Depending on the magnitude, diastolic SR Ca^2+^ leak through *RYR2* can be categorized as Ca^2+^ waves, Ca^2+^ sparks, and nonspark Ca^2+^ leak, all of those are proarrhythmic for different reasons. Ca^2+^ waves are large and cell‐wide SR Ca^2+^ release events that initiates from spontaneous opening of *RYR2*s in one *RYR2* cluster activating neighboring *RYR2* clusters and causing propagating Ca^2+^ release events. Ca^2+^ waves promote large inward depolarizing current via NCX1 that may lead to DADs.^[^ ^48^ ^]^ Ca^2+^ sparks were discovered as the elementary SR Ca^2+^ release event,^[^ ^49^ ^]^ which can be detected by confocal microscopy with the use of a Ca^2+^‐sensitive fluorescent dye. However, later it is clear that Ca^2+^ sparks are not “elementary” since some SR Ca^2+^ release events are beyond detection by line‐scanning confocal microscopy, and increasing evidence suggest *RYR2*‐mediated SR Ca^2+^ leak in nonspark form or “silent” leak contributes significantly to overall SR Ca^2+^ leak during diastole,^[^ ^46^, ^50^, ^51^, ^52^ ^]^ and smaller clustering of *RYR2*s could be a major mechanism underlying the silent leak.^[^ ^53^, ^54^ ^]^ While it was traditionally believed that junctional SR in cardiomyocytes are uniformly packed with *RYR2*s, super‐resolution microscopy studies have indicated that junctional SR is in fact composed of multiple *RYR2* clusters that vary in size and morphology,^[^ ^18^, ^53^, ^55^, ^56^, ^57^ ^]^ with significant amount of small *RYR2* clusters. It was reported that 42% *RYR2* clusters contained five or fewer *RYR2*s in ventricular myocytes of WT mice.^[^ ^53^ ^]^ In our study, this number was ≈33% in control iPSC‐CMs, but was increased to ≈50% in ARVC iPSC‐CMs. For this particular reason, tetracaine protocol instead of Ca^2+^ spark was selected to assess total *RYR2*‐mediated SR Ca^2+^ leak. Indeed, the total leak was significantly augmented in ARVC iPSC‐CMs compared to both control and GC iPSC‐CMs.

It has been recently reported that *TMEM43* localized at the NE in mouse cardiomyocytes.^[^ ^6^, ^19^ ^]^ However, its exact sublocalization in human cardiomyocytes remained unclear. We showed that *TMEM43* localized at both NE and cytoplasm in control iPSC‐CMs, and the P386S mutation led to translocalization of *TMEM43* from the NE to the cytoplasm. Notably, a striking finding that was previously undescribed was the dramatic change of the arrangement of *TMEM43* localized at cytoplasmic region in P386S iPSC‐CMs. These changes of *TMEM43* localization and arrangement by P386S were associated with the abnormal and wrinkled NE, which were consistent with the previous evidence that *TMEM43* associates with LINC complex components to maintain the nuclear structure.^[^ ^8^, ^58^, ^59^ ^]^

We identified *TMEM43* interacts with lamin B2 in both control and mutant iPSC‐CMs, while TMEM43‐P386S did not abolish the interaction between *TMEM43* and lamin B2, but led to mislocalization of lamin B2 at the nucleus. Nuclear lamina forms a fibrous meshwork lining the inner nuclear membrane, not only provides scaffolding for the nuclear envelope, an attachment site for chromatin and an environment that promotes gene inactivation, but also plays essential roles in regulating signaling and gene activity as well as in mechanically coupling the cytoplasmic cytoskeleton to the nucleus.^[^ ^8^, ^60^, ^61^ ^]^ Indeed, lamins, including lamin A, B1, B2, and C, are major components of nuclear lamina that are highly involved in DNA replication, organization of chromatin, mechanical stabilization of the nucleus, positioning of nuclear pores, and anchoring of nuclear membrane components.^[^ ^20^ ^]^ Hence, we reasoned that mislocalization of lamin B2 conferred the abnormal NE structure in ARVC iPSC‐CMs. Consequently, our ATAC‐Seq data revealed that the global chromatin conformation was disrupted, leading to decreased opening of promoters around downregulated genes, including *RYR2*.

To investigate the in vivo effects of the TMEM43‐P386S mutation, we generated TMEM43‐P386S KI mice. As anticipated, these KI mice exhibited arrhythmias when subjected to ISO/Caff stimulation, with the homozygous mice experiencing more severe arrhythmias than heterozygous one. Remarkably, the administration of flecainide prior to ISO/Caff stimulation effectively reversed the aforementioned arrhythmias in both homozygous and heterozygous TMEM43‐P386S KI mice, highlighting the potential of flecainide in preventing stress‐induced cardiac arrhythmias in these mice. Furthermore, we extended our investigation to cellular level to further elucidate the rescue effect of flecainide in vivo. Our findings revealed a significant increase in SCR following pacing in KI mice compared to WT mice. Importantly, flecainide demonstrated its ability to effectively alleviate the SCR events observed in KI mice. This was evident in both the reduced incidence of SCR events and the decreased percentage of cells exhibiting SCR in response to flecainide treatment. These results highlight flecainide's potential in ameliorating abnormal cellular Ca^2+^ handling associated with the TMEM43‐P386S mutation.

The traditional cardiomyopathy models based on immortalized cell lines may not accurately replicate pathological phenotypes, while iPSC‐CMs offer a model that more closely mimics human physiological characteristics. However, limitations still exist. iPSC‐CMs do not fully possess the traits of mature cardiomyocytes, which limits their application in disease modeling and drug screening. The maturation process of cardiomyocytes is controlled by a precise transcriptional regulatory program, which iPSC‐CMs may not fully replicate in vitro. By delving into the maturation process and regulatory mechanisms of cardiomyocytes, the preparation methods of iPSC‐CMs can be optimized to better mimic the functions of mature cardiomyocytes, thereby enhancing the accuracy of cardiovascular disease modeling and drug screening.

In conclusion, both iPSC‐CMs and KI mice recapitulate arrhythmic features associated with *TMEM43*‐related ARVC. We provide robust evidence for the causal role of TMEM43‐P386S in ARVC. We find decreased *RYR2* cluster size and hyperphosphorylated *RYR2*, which could be the underlying mechanisms leading to hyperactive *RYR2*, Ca^2+^ mishandling, and subsequent arrhythmias in our case (Figure 8I). We anticipate that these findings will help uncover the molecular mechanisms underlying cardiac arrhythmogenesis in *TMEM43*‐related ARVC and may facilitate the development of novel therapeutic approaches for this condition.

## 4 Experimental Section

### Patient Recruitment

A 3 mm skin punch biopsy was taken from the patient with ARVC following informed consent. This study conformed to the principles in the Declaration of Helsinki and was approved by the Ethics Committee of the First Affiliated Hospital, Zhejiang University School of Medicine (IIT20250034B, [2025B] IIT Ethics Approval No. 0102).

### Genetic Testing

Peripheral blood sample was drawn from the ARVC patient and sent to MyGenostics Inc. (Beijing, China) for isolation of genomic DNA and commercial genetic testing. Genomic DNA of 1–3 µg was fragmented to an average size of 150 bp using a S220 Focused‐ultrasonicator (Covaris). A DNA Sample Prep Reagent Set (MyGenostics Inc.) was used for the preparation of standard libraries (including end repair, adapter ligation, and PCR amplification), which were further sequenced by Illumina NovaSeq6000. The amplified DNA was captured using GenCap cardiomyopathy capture kit (MyGenostics Inc.). The gene panel for cardiomyopathy included the following genes: *AARS2*, *ABCC9*, *ACTC1*, *ACTN2*, *AKAP9*, *ANK2*, *ANKRD1*, *BAG3*, *BRAF*, *CACNA1C*, *CACNB2*, *CALR3*, *CASQ2*, *CAV3*, *CBL*, *CRIP3*, *CRYAB*, *CSRP3*, *DES*, *DMD*, *DSC2*, *DSG2*, *DSG3*, *DSP*, *DTNA*, *EMD*, *EYA4*, *FHL2*, *FHOD3*, *FKTN*, *GATAD1*, *GLA*, *GPD1L*, *HCN4*, *ILK*, *JPH2*, *JUP*, *KCNE1*, *KCNE2*, *KCNE3*, *KCNH2*, *KCNJ5*, *KCNQ1*, *KRAS*, *LDB3*, *LMNA*, *MIB1*, *MYBPC3*, *MYH6*, *MYH7*, *MYL2*, *MYL3*, *MYLK2*, *MYOZ2*, *MYPN*, *NEXN*, *NRAS*, *OBSCN*, *PDLIM3*, *PKP2*, *PLN*, *PRDM16*, *PRKAG2*, *PSEN1*, *PSEN2*, *PTPN11*, *RAF1*, *RBM20*, *RYR2*, *SCN1B*, *SCN3B*, *SCN4B*, *SCN5A*, *SCO2*, *SGCD*, *SHOC2*, *SLC25A4*, *SNTA1*, *SOS1*, *TAZ*, *TCAP*, *TGFB3*, *TMEM43*, *TMEM70*, *TMPO*, *TNNC1*, *TNNI3*, *TNNT2*, *TNNT3*, *TPM1*, *TTN*, *TTR*, *VCL*.

### Culture and Maintenance of Skin Fibroblasts

Freshly isolated skin biopsies were rinsed with Dulbecco's phosphate‐buffered saline (DPBS) (Gibco, C14190500BT) and transferred into a 1.5 mL tube. Tissue was minced in collagenase I (1 mg mL^−1^ in Dulbecco's modified Eagle medium (DMEM), Gibco, C11995500BT) and allowed to digest for 6 h at 37 °C. Dissociated skin fibroblasts were plated and maintained with DMEM containing 10% fetal bovine serum (Gibco, 10091148) and Penicillin–Streptomycin (Gibco, 15140122) at 37 °C, 95% air, and 5% CO_2_ in a humidified incubator. All skin fibroblasts were used for reprogramming within 5 passages.

### Generation of iPSC Lines

Somatic reprogramming was used to generate ARVC patient‐specific iPSC lines from skin fibroblasts using CytoTune‐iPS 2.0 Sendai Reprogramming Kit following the manufacturer's instructions (Invitrogen, A16517).

### Culture and Maintenance of iPSCs

The iPSCs were cultured in feeder‐free mTeSR1 (STEMCELL Technologies, 85850) media on matrigel (Corning, 354277)‐coated plates at 37 °C with 5% (vol/vol) CO_2_. The media were daily changed and iPSCs were passaged every 3–4 days using Accutase (STEMCELL Technologies, 07920), and resuspended and seeded in mTeSR1 containing 10 µm Y27632 ROCK inhibitor (Selleck, S1049).

### Genetic Sequencing at the iPSC Level

The iPSCs were cultured on matrigel‐coated 6‐well plates with mTeSR1 and harvested at 80–90% confluence for subsequent analysis. Genomic DNA was extracted using a commercial DNA isolation kit (TIANGEN, DP304‐03). PCR was carried out on EasyCycler 96 (Analytik Jena). Single SNP loci within the *TMEM43* gene (c.1156C>T; p.P386S) was amplified and analyzed by direct sequencing and confirmed by subcloning. The forward and reverse primer sequences for *TMEM43* were 5′‐TGGTTTCCTGTTTTCCGAGACCT‐3′ and 5′‐GCCGTACCCACTCAGTCCA‐3′, respectively.

### Karyotyping

Chromosome analysis by G‐banding was achieved using iPSCs at passage 20 by the Prenatal Diagnosis Center of Hangzhou Women's Hospital. At least 20 metaphase cells were analyzed at 300–400 band level.

### Alkaline Phosphatase Staining

ALP staining was performed using the VECTOR Blue Alkaline Phosphatase Substrate Kit (Vector Laboratories, SK‐5300) following the manufacturer's instructions.

### Teratoma Formation

5 weeks old female NOD/SCID mice were purchased (GemPharmatech) and utilized for teratoma formation assay. Approximately 1 × 10^6^ iPSCs at passage 20 were digested using Accutase and suspended in 0.5 mL mTeSR1 supplemented with 10 µm Y27632. After mixed with 0.5 mL matrigel, the cells were injected into the armpits and back of the mice. 4–6 weeks after cell delivery, teratomas were dissected and fixed with 4% paraformaldehyde (PFA) (Beyotime Biotechnology, P0099) in phosphate buffered saline (PBS) (Sangon Biotech, B040100) for hematoxylin and eosin (H&E) staining.

### Gene Correction of TMEM43‐P386S in ARVC iPSCs by CRISPR/Cas9

The exon 12 of *TMEM43* was selected for guide RNA (gRNA) design and the gRNA was designed according to the CRISPR online design tool: http://crispor.tefor.net. The sequences of a pair of oligos for targeting site were listed as followed: forward, 5′‐ CACCGCATCCTTGTTGCTCGGACAC‐3′; reverse, 5′‐ AAACGTGTCCGAGCAACAAGGATGC‐3′. The oligos were annealed and ligated into linearized lentiCRISPRv2 vector for generating gRNA‐expressing plasmid. 1 × 10^5^ ARVC iPSCs were seeded in 12‐well plates to 80% confluence. The recombinant plasmid and single‐stranded oligo deoxynucleotide (ssODN) as repair template were transfected into ARVC iPSCs using Lipofectamine 3000 Transfection Reagent (Invitrogen, L3000‐015) according to the manufacturer's instructions. The cells were placed in mTeSR1 supplemented with puromycin (Gibco, A1113803) at a concentration of 4 µg mL^−1^. After 2–3 days, puromycin‐resistant clones were picked and then verified by genomic PCR and DNA sequencing (Sangon Biotech).

### KI of TMEM43‐P386S in Control iPSCs by CRISPR/Cas9

The exon 12 of *TMEM43* was selected for gRNA design and the gRNA was designed according to the CRISPR online design tool: http://crispor.tefor.net. The sequences of a pair of oligos for targeting site were listed as followed: forward, 5′‐ CACCGCATCCTTGTTGCTCGGACAC‐3′; reverse, 5′‐ AAACGTGTCCGAGCAACAAGGATGC‐3′. The oligos were annealed and ligated into linearized lentiCRISPRv2 vector for generating gRNA‐expressing plasmid. 1 × 10^5^ control #2 iPSCs were seeded in 12‐well plates to 80% confluence. The recombinant plasmid and ssODN as mutation template were transfected into control #2 iPSCs using Lipofectamine 3000 Transfection Reagent. The cells were placed in mTeSR1 supplemented with puromycin at a concentration of 4 µg mL^−1^. After 2–3 days, puromycin‐resistant clones were picked and then verified by genomic PCR and DNA sequencing.

### Cardiac Differentiation

The iPSC‐CMs were generated using a 2D monolayer differentiation protocol. Briefly, ≈10^5^ undifferentiated iPSCs were dissociated and replated into matrigel‐coated 6‐well plates. The iPSCs were cultured and expanded to 85% confluence, and then treated for 2 days with 6 µm CHIR99021 (Axon Medchem, 1386) in RPMI 1640 (Gibco, C11875500BT) with B27 supplement minus insulin (Gibco, A1895601) (RPMI + B27 − Insulin) to activate Wnt signaling pathway. On day 2, cells were placed in RPMI + B27 − Insulin with CHIR99021 removal. On days 3–4, cells were treated with 5 µm IWR‐1 (Millipore, 681669) to inhibit Wnt signaling pathway. On days 5–6, cells were removed from IWR‐1 treatment and placed in RPMI + B27 − Insulin. From day 7 onward, cells were placed and cultured in RPMI 1640 and B27 supplement with insulin (Gibco, 17504044) (RPMI + B27 + Insulin) until beating was observed. Cells were glucose‐starved for 3 days with RPMI + B27 + Insulin for the purification. Cardiomyocytes of days 30–40 after cardiac differentiation were utilized for downstream functional assays.

### Immunofluorescent Staining

Cells were fixed with 4% PFA for 15 min, permeabilized with 0.1% Triton X‐100 (Sangon Biotech, A110694) for 5 min, and blocked with 3% bovine serum albumin (Sigma‐Aldrich, A1933) for 1 h. Cells were subsequently stained with appropriate primary antibodies and AlexaFluor conjugated secondary antibodies. Primary antibodies included OCT4 (Cell Signaling Technology, 2750S, 1:200), NANOG (Santa Cruz Biotechnology, sc‐33759, 1:200), SSEA‐4 (Abcam, ab16287, 1:200), SOX2 (Abcam, ab171380, 1:200), *TNNT2* (Abcam, ab8295, 1:500), α‐actinin (Cell Signaling Technology, 6487P, 1:100), FLAG (Sigma‐Aldrich, F1804, 1:500), lamin A/C (Abcam, ab108595, 1:400), lamin B1 (Abcam, ab133741, 1:200), lamin B2 (Cell Signaling Technology, 12255S, 1:50), emerin (Santa Cruz Biotechnology, sc‐25284, 1:50), and *RYR2* (Abcam, ab2868, 1:100). Secondary antibodies included AlexaFluor 647 (Abcam, ab150079, 1:200), AlexaFluor 594 (Abcam, ab150080, 1:500; Abcam, ab150108, 1:500), and AlexaFluor 488 (Abcam, ab150113, 1:500; Invitrogen, A11008, 1:500). Nuclei were stained with DAPI (Roche Diagnostics, 1023276001, 1 µg mL^−1^). Pictures were taken with 60× objective on confocal microscope (Nikon, A1) using NIS‐Elements AR software (Nikon).

### Patch Clamp Recordings from iPSC‐CMs

The iPSC‐CMs were mechanically and enzymatically dissociated to obtain single cells, which were seeded on matrigel‐coated glass coverslips (Warner Instruments). Cells with spontaneous beatings were selected and action potentials were recorded using an EPC‐10 patch clamp amplifier (HEKA). Continuous extracellular solution perfusion was achieved using a rapid solution exchanger (Bio‐logic Science Instruments, RSC‐200). All signals were acquired using PatchMaster software (HEKA) and filtered at 1 kHz and digitized at 10 kHz. Data analyses were performed using Igor Pro (Wavemetrics) and GraphPad Prism (GraphPad Software). A TC‐344C dual channel heating system (Warner Instruments) was used to maintain the temperature at 35.5–37 °C. Tyrode's solution was used as the external solution containing 140 mm NaCl, 5.4 mm KCl, 1 mm MgCl_2_, 10 mm glucose, 1.8 mm CaCl_2_, 1.0 mm Na‐Pyruvate and 10 mm *N*‐(2‐hydroxyethyl)piperazine‐29‐(2‐ethane‐sulfonic acid) (HEPES) (pH 7.4 with NaOH). The internal solution contained 140 mm KCl, 5.0 mm NaCl, 10 mm HEPES, 5 mm Mg‐ATP, and 5 mm ethylene glycol‐bis(*b*‐aminoethylether)‐*N*,*N*,*N9*,*N9*‐tetraacetic acid (EGTA) (pH 7.2 with KOH). Key action potential parameters were quantified, including MDP, overshoot, APA, action potential duration at 50% and 90% repolarization (APD_50_ and APD_90_), maximal upstroke velocity (*V*_max_), beating rate, and SD of interspike intervals (ISIs). Ventricular‐like iPSC‐CMs were distinguished based on the action potential morphology and action potential parameters, which exhibited a clear plateau phase, larger APA and *V*_max_ values, more negative MDP values, APD_30–40_/APD_70–80_ > 1.5, and APD_90_/APD_50_ ≤ 1.3.

### MEA

For cell preparation, a 20 µL droplet of coating solution (matrigel) was applied on the area of the electrodes of the MEA probes (Multi Channel Systems, 60MEA200/30iR‐Ti‐gr), which were incubated at 37 °C in 5% CO_2_ for at least 1 h. The iPSC‐CMs were then dissociated from the 6‐well plates using TrypLE (Gibco, 12604013), and reseeded onto MEA probes at a density of 1–1.5 × 10^5^ cells in 20 µL bead of the cell suspension per well. After incubation with 5% CO_2_ at 37 °C for 1 h to promote adhesion, each MEA probe was filled with culture media to a final volume. Culture medium was changed after 2 days, and afterward the medium was changed every 2 or 3 days throughout 5–7 days of culturing period. Field potentials were recorded from spontaneously beating iPSC‐CMs using the MEA2100 data acquisition system (Multi Channel Systems) with sampling at 10 kHz. All experiments were performed at 37 °C and began after a 20 min equilibration period. The data were analyzed with Cardio 2D^+^ software (Multi Channel Systems) and steady‐state parameters were averaged.

### Ca^2+^ Imaging Using Fluo‐4 AM

The iPSC‐CMs grown on coverslips were loaded with RPMI 1640 medium without Phenol Red (Gibco, 11835030) supplemented with 5 µm Fluo‐4 AM (Invitrogen, F23917) for 15 min in the dark at room temperature. After washing with prewarmed DPBS and RPMI 1640 for 2 times, the iPSC‐CMs were then immersed in imaging buffer for 30 min before imaging experiments. For imaging, iPSC‐CMs were placed in a chamber equipped with a temperature controller under constant perfusion of 37 °C imaging buffer. Ca^2+^ signaling was made by recording the fluorescence of cells using an Ultra High Speed Wavelength Switcher (Lambda DG‐4, Sutter Instruments) with a CCD camera (Zyla, Andor) mounted on an inverted microscope (Eclipse Ti, Nikon). Data were acquired using NIS‐Elements software (Nikon) and were quantified as the extracellular background signal subtracted fluorescence intensity (*F*) and then normalized to the baseline fluorescence (*F*_0_). Transient amplitude was presented as a relative scale (Δ*F*/*F*_0_).

### Ca^2+^ Imaging Using Fura‐2 AM

The iPSC‐CMs grown on coverslips were loaded with RPMI 1640 medium without phenol red (Gibco, 11835030) supplemented with 5 µm Fura‐2 AM (Invitrogen, F14185) for 15 min in the dark at room temperature. After washing with prewarmed DPBS and RPMI 1640 for 2 times, before experiment the cells were immersed in imaging buffer for 30 min. Ca^2+^ signaling was made by recording the fluorescence of cells using an Ultra High Speed Wavelength Switcher (Lambda DG‐4, Sutter Instruments) with a CCD camera (Zyla, Andor) mounted on an inverted microscope (Eclipse Ti, Nikon). Data were acquired using NIS‐Elements software (Nikon). Fluorescent signals were obtained upon excitation at 340 (*F*_340_) and 380 nm (*F*_380_). Amplitude of Ca^2+^ transient was defined as the ratio of *F*_340_/*F*_380_.

### Assessment of *RYR2*‐Mediated Diastolic Ca^2+^ Leak and SR Ca^2+^ Load

*RYR2*‐mediated diastolic Ca^2+^ leak and SR Ca^2+^ load was assayed using Fluo‐4 fluorescence. The iPSC‐CMs were incubated for 15 min at room temperature in phenol red‐free RPMI 1640 (Gibco, 11835030) containing 5 µm of the cytosolic Ca^2+^ dye Fluo‐4 AM (Invitrogen, F23917) to load the indicator into the cytosol. Following incubation, the indicator‐containing medium was removed, and iPSC‐CMs were washed with phenol red‐free RPMI 1640 for 3 times and immersed in imaging buffer for an additional 30 min at room temperature to allow for de‐esterification of the indicator. Recordings were captured using an epifluorescence microscope. The iPSC‐CMs were field‐stimulated at 1 Hz in normal Tyrode's solution containing 1.8 mm Ca^2+^ until achieving a steady‐state intracellular Ca^2+^ content. The stimulation was then turned off, and the bath solution was rapidly changed to 0 Na^+^, 0 Ca^2+^ Tyrode's solution (140 mm LiCl, 5.4 mm KCl, 1 mm MgCl_2_, 5 mm HEPES, 10 mm glucose, 10 mm EGTA, pH 7.4 with LiOH) to eliminate trans‐sarcolemmal Ca^2+^ fluxes. A pulse of tetracaine (1 mm) was added to inhibit *RYR2*. The observed shift (Ca^2+^ from cytosol to SR) was proportional to SR Ca^2+^ leak. Solution was then rapidly switched to caffeine (10 mm) to deplete SR Ca^2+^ stores. Finally, bath solution was switched from 0 Na^+^, 0 Ca^2+^ Tyrode's solution back to normal Tyrode's solution. Data were acquired using NIS‐Elements software (Nikon Instruments Inc.), and analysis was performed using Igor Pro (Wavemetrics) and GraphPad Prism (GraphPad Software).

### Transmission Electron Microscopy

The iPSC‐CMs were dissociated with Tripsin–ethylenediaminetetraacetic acid (EDTA) (Gibco, 25200072), scrapped into a 1.5 mL microcentrifuge tube and centrifuged, and then fixed with 2.5% glutaraldehyde (Sigma‐Aldrich, G5882) in 0.1 m phosphate buffer overnight at 4 °C. The specimen was postfixed with 1% OsO_4_ in phosphate buffer and dehydrated by a graded series of ethyl‐alcohol for 15–20 min at each step, then transferred to absolute acetone for 20 min. Next, the specimen was placed in 1:1 mixture of absolute acetone and final spur resin mixture for 1 h at room temperature, and transferred to 1:3 mixture of absolute acetone and final spur resin mixture for 3 h, and then transferred to final spur resin mixture overnight. The specimen was subsequently placed in 1.5 mL tube contained spur resin, heated at 70 °C for more than 9 h, and sectioned using a LEICA EM UC7 ultratome. The sections were then stained with uranyl acetate and alkaline lead citrate for 5–10 min. Pictures were observed using a transmission electron microscopy (Hitachi, Model H‐7650).

### Tau‐STED Super‐Resolution Imaging

Tau‐STED super‐resolution imaging was performed on a Leica Stellaris 8 Tau‐STED microscope, using an HC PL APO CS2 100×/1.40 oil immersion objective lens (Leica Microsystems). Emission depletion was accomplished with a 775 nm STED laser. Excitation was provided by a white light laser at the desired wavelength for each sample.

AlexaFluor 594 was excited at 561 nm and its fluorescence emission was detected at 570–620 nm using a hybrid detector (Leica Microsystems). 1024 × 1024 pixel images were acquired with a pixel size of 14 nm.

### Overexpression of *TMEM43* in iPSC‐CMs

The TMEM43‐P386S mutation was generated by site‐directed mutagenesis using the Mut Express II Fast Mutagenesis Kit V2 (Vazyme) from human WT *TMEM43* cDNA. WT or mutant *TMEM43* fragment was subcloned into the GFP‐carrying vector (pCDH‐CMV‐MCS‐EF1‐copGFP). The recombinant lentiviral vector and the packaging plasmids psPAX2 and pMD2.G were transfected into human embryonic kidney 293T cells using Lipofectamine 3000 to produce the lentivirus, and virus was harvested 3–4 days after the transfection. The harvested virus was filtered using a 0.45 µm Cell Strainer (Millipore) and was concentrated by 5× PEG8000 (MKBio, MS4323). Control iPSC‐CMs were grown in 12‐well plates to 80% confluence, and were then transfected with purified virus according to the virus titer. Polybrene (5–10 µg mL^−1^) (Sigma‐Aldrich) was added in parallel to enhance the efficiency of transfection. Multiplicity of infection for transfection of the iPSC‐CMs was optimized to levels at which GFP could be detected in 80–90% cells.

### Real‐Time Quantitative PCR

Mouse heart tissues were ground in TRIzol Reagent (Life Technologies, 15596018CN) for RNA extraction. cDNA was synthesized using the 4 × EZscript Reverse Transcription Mix II (EZBioresearch, EZB‐RT2GQ). qPCR was performed using SYBR Green PCR Master Mix (Takara, RR420A). mRNA expression levels were normalized to the housekeeping gene *Gapdh*, which served as an internal control for gene expression studies. The specific primer sequences for *Ryr2* were 5′‐ACGGCGACCATCCACAAAG‐3′ and 5′‐AAAGTCTGTTGCCAAATCCTT‐3′, while those for *Atp2a2* were 5′‐TGGAACAACCCGGTAAAGAGT‐3′ and 5′‐CACCAGGGGCATAATGAGCAG‐3′.

### Western Blot

The iPSC‐CMs were detached with TrypLE and then pelleted at 1000 rpm for 5 min at 4 °C. After washing with PBS, the pellets were resuspended in 50–100 µL lysis buffer. Mouse heart tissue was ground after adding lysis buffer. Lysates were placed on ice for 30 min and then the supernatants were collected after centrifuging at 12 000 rpm for 15 min. Cytosolic and nuclear fractions were extracted with nuclear and cytoplasmic protein extraction kit (Beyotime Biotechnology, P0027) according to the manufacturer's instruction. Protein concentration was measured using a BCA kit (Pierce, 23227). Western blot was performed using standard protocol with the following antibodies: Ca_v_1.2 (ProteinTech, 21774‐1‐AP, 1:500), NCX1 (ProteinTech, 55075‐1‐AP, 1:1000), total *PLN* (Cell Signaling Technology, 14562S, 1:1000), *SERCA2a* (Santa Cruz Biotechnology, sc‐53010, 1:200), total *RYR2* (Abcam, ab2827, 1:500), phosphorylated *RYR2* (Ser2814) (Badrilla, Aolo‐3/AP, 1:500), GAPDH (Abmart, M20006M, 1:5000), *TMEM43* (Santa Cruz Biotechnology, 365298, 1:400; Abcam, 184164, 1:2000), α‐tubulin (Beyotime Biotechnology, AF0001, 1:1000), PARP (Cell Signaling Technology, 9532, 1:1000), lamin A/C (Abcam, ab108595, 1:1000), lamin B1 (Abcam, ab133741, 1:500), lamin B2 (Abcam, ab151735, 1:500), and emerin (Santa Cruz Biotechnology, sc‐25284, 1:200). Intensity values for each band were determined as the integrated density (sum of pixel values) within a fixed area using Quantity One software (Biorad).

### RNA‐Seq

The transcriptome library for sequencing was generated using VAHTSTM mRNA‐seq v2 Library Prep Kit for Illumina (Vazyme Biotech) following the manufacturer's recommendations. The libraries were next sequenced on an Illumina HiseqXTen platform using the (2 × 150 bp) paired‐end module. Reads with adapters and low‐quality reads (>50% of bases with quality ≤ 10% or >5% of unknown bases) were removed. The clean reads obtained were aligned to the human reference genome GRCh38 using HISAT2 (v2.2.1). The resulting alignment files were then processed by Cufflinks software (v2.2.1) for the estimation of gene expression abundance. DEGs were identified using the Cuffdiff program between GC and ARVC iPSC‐CMs, with a criterium of a more than twofold change in expression and adjusted *p* value < 0.05. GO enrichment analysis and KEGG pathway enrichment analysis were both performed using clusterProfiler (v4.10.1).

### ATAC‐Seq

Samples with 5 × 10^5^ iPSC‐CMs were shipped on dry ice to Novogene (Beijing, China). All subsequent processing was performed by Novogene. Briefly, nuclei were extracted from samples and the nuclei pellet was resuspended in the Tn5 transposase reaction mix. The transposition reaction was incubated at 37 °C for 30 min. Equimolar Adapter 1 and Adapter 2 were added after transposition, and then PCR was performed to amplify the library. After the PCR reaction, libraries were purified with the AMPure beads and library quality was assessed with Qubit. The ATAC‐seq libraries were sequenced with the NovaSeq6000 sequencing platform at Novogene. Nextera adaptor sequences were first trimmed from the reads using skewer (0.2.2). These reads were aligned to a reference genome using BWA with standard parameters. These reads were then filtered for high quality (MAPQ ≥ 13), nonmitochondrial chromosome, and properly paired reads (longer than 18 nt). All peak calling was performed with macs2 using “macs2 callpeak –nomodel –keepdup all –call‐summits.” For simulations of peaks called per input read, aligned and deduplicated BAM files were used without any additional filtering.

### Co‐IP

The iPSC‐CMs were lysed with lysis buffer (50 mm Tris/HCl, 150 mm NaCl, 1% Triton X‐100, 1 mm EDTA, 10% glycerin, and Protease Inhibitor Cocktail (Roche, 04693116001), pH 7.4) and incubated for 30 min in 4 °C rotation. The lysates were then centrifuged at 13 000 *g* for 10 min to yield the protein extract in the supernatant. One fraction containing 0.5 mg of protein was incubated with 2 µg antibody and 20 µL of beads overnight at 4 °C to immunoprecipitate proteins; the other fraction was prepared as input protein. After incubation, immunoprecipitated proteins were washed 3 times with washing buffer. The input and immunoprecipitated proteins were both subjected to Western blot analysis.

### Mass Spectrometry (MS) Analysis for *TMEM43*‐Interacting Proteins

To identify *TMEM43*‐interacting proteins, the co‐immunoprecipitated proteins were pull‐down through Pierce Protein A/G Magnetic Beads (Pierce, 88804) after separation by electrophoresis using Commassie brilliant blue (CBB) Fast Staining Solution (TIANGEN, PA101) following the manufacturer's protocol. The CBB‐stained gel spots were excised and sent to OE Biotech (Shanghai, China) for liquid chromatography–mass spectrometry analysis using a mass spectrometer (Thermo Scientific, Q Exactive). Protein gel bands were cut into small pieces and destained with destaining buffer (25 mm NH_4_HCO_3_/25% methanol, pH 8.0). Gel pieces were reduced with 10 mm dithiothreitol for 60 min at 56 °C and alkylation with 55 mm iodoacetamide for 45 min. Gel pieces were washed with digestion buffer (50 mm NH_4_HCO_3_, pH 8.0) twice, dehydrated with acetonitrile, and the sample was then dried with speed vac. Gel pieces were rehydrated with trypsin solution (10 ng L^−1^ sequencing grade modified trypsin, 50 mm NH_4_HCO_3_, pH 8.0) and incubated overnight at 37 °C. Digested peptides were sequentially extracted from gel pieces with elution buffer 1 (50% acetonitrile, 5% formic acid), elution buffer 2 (75% acetonitrile, 0.1% formic acid). Gel pieces were dehydrated with acetonitrile twice, and all the supernatants were combined. Peptide solution was dried with speed vac, and digested peptides were resuspended with 5% formic acid and desalted with StageTip.

The evaporated peptide samples were redissolved in buffer A (99.9% H_2_O, 0.1% acetic acid) and loaded on trap column by autosampler. Peptides were separated by analysis column (Thermo Scientific, 75 µm × 150 mm RP‐C18) at 300 nL min^−1^ flow rate and 30 min mobile phase gradient with EASY‐nLC 1200 high‐performance liquid chromatography system. Q Exactive HF‐X mass spectrometer was operated in data‐dependent mode with one full MS scan *R* = 70 000 followed by 10 HCD MS/MS scan at *R* = 17 500. The AGC targets for MS1 and MS2 scans were 3 × 10^6^ and 5 × 10^5^, respectively, and the maximum injection time for MS1 and MS2 were 50 and 50 ms, respectively. Dynamic exclusion was set to 40 s, and mass spectrometry data were searched by MaxQuant.

### Generation of Lamin B2 Knockout iPSC Lines by CRISPR/Cas9

The exon 1 of *LMNB2* was selected for gRNA design and the gRNA was designed according to the CRISPR online design tool: http://crispor.tefor.net. The sequences of a pair of oligos for targeting site were listed as followed: forward, 5′‐ CACCGTGTCGCCCACGCGCCTGTCG‐3′; reverse, 5′‐ AAACCGACAGGCGCGTGGGCGACAC‐3′. The oligos were annealed and ligated into linearized lentiCRISPRv2 vector for generating gRNA‐expressing plasmid. 1 × 10^5^ control iPSCs were seeded in 12‐well plates to 80% confluence. The recombinant plasmid was transfected into control iPSCs using Lipofectamine 3000. The cells were placed in mTeSR1 supplemented with puromycin at a concentration of 4 µg mL^−1^. After 2–3 days, puromycin‐resistant clones were picked and verified by genomic PCR and DNA sequencing (Sangon Biotech).

### Generation of TMEM43‐P386S KI Mice

TMEM43‐P386S KI mice (Strain No. GJS021905955‐GPS00002917) were generated using the CRISPR/Cas9 approach (GemPharmatech, Nanjing, China). First, one sgRNA‐targeting *Exon12* of *Tmem43* was, respectively, constructed and transcribed in vitro. And the donor vector with the TCC fragment was designed and constructed in vitro. Then, Cas9 mRNA, sgRNA, and donor were coinjected into zygotes. Thereafter, the zygotes were transferred into the oviduct of pseudopregnant ICR females at 0.5 dpc. And F0 mice was birthed after 19–21 days of transplantation, all the offsprings of ICR females (F0 mice) were identified by PCR and sequencing of tail DNA. And positive F0 mice which had a copy of the point mutation of *TMEM43* were genotyped by the methods. Finally, crossing F0 mice with C57BL/6J mouse to build up heterozygous mice. All animal‐related experimental procedures and methodologies were approved by the Animal Experimental Ethical Inspection of the First Affiliated Hospital, Zhejiang University School of Medicine (2024‐643).

### Electrophysiological Analysis

Mice were anesthetized with isoflurane vapor (0.5–1%). Electrode needles were inserted subcutaneously into the left, right upper limb and right lower limb for ECG recording (iWorx system, IX‐RA‐B3G). The ECG waveforms were monitored until a stable baseline and heart rate were reached. Baseline ECG was recorded for 2 min, followed by the intraperitoneal injection of isoprenaline (2 mg kg^−1^) and caffeine (120 mg kg^−1^). ECG was then continuously recorded for another 30 min. ECG traces were analyzed using the LabScribe 4.361 (iWorx system). To test the efficacy of flecainide in vivo, 5% DMSO was first administered intraperitoneally in WT and KI (heterozygous and homozygous) mice 15 min before ISO/Caff stress, and ECG was recorded for 30 min. 2–3 days later, flecainide (20 mg kg^−1^) was administered by the intraperitoneal injection in the aforementioned mice 15 min before ISO/Caff stress, followed by 30 min ECG recordings.

### Histological Analysis

Mice were subjected to histological analysis using H&E, Masson's trichrome, and Oil Red O stains. Hearts were collected immediately after sacrifice and fixed in 4% PFA for 24 h at 4 °C. Subsequently, the tissues were embedded in paraffin or Tissue‐Tek O.C.T. Compound (SAKURA, 4583) and cut into 5 µm thick sections for further experiments. The sections were stained with H&E (Servicebio, G1003), Masson's trichrome (Servicebio, G1006), or Oil Red O (MKbio, MS4009) to assess heart morphology, cardiac fibrosis, and lipid deposition. All sections were visualized under a microscope (Olympus, VS200).

### Measurement of Spontaneous SR Ca^2+^ Release in Isolated Mouse Ventricular Myocytes

The ventricular myocytes isolated from adult mouse hearts were loaded with normal Tyrode's solution containing 1.8 mm Ca^2+^ supplemented with 5 µm Fluo‐4 AM for 15 min in the dark at room temperature. After washing with dye‐free normal Tyrode's solution for 30 min, cells were then placed in a chamber equipped with a pair of parallel electrodes under constant perfusion of imaging buffer at room temperature. Ca^2+^ signaling was made by recording the fluorescence of cells using an Ultra High Speed Wavelength Switcher with a CCD camera mounted on an inverted microscope. Data were acquired using NIS‐Elements software (Nikon) and were quantified as the extracellular background signal subtracted fluorescence intensity (*F*) and then normalized to the baseline fluorescence (*F*_0_). Transient amplitude was presented as a relative scale (Δ*F*/*F*_0_). After being paced from 1 to 2 Hz to baseline stability, only myocytes demonstrating clear striation and normal contractility were chosen for further analysis. When steady‐state Ca^2+^ transient was obtained, pacing was paused for 30 s to record spontaneous SR Ca^2+^ release.

### Ca^2+^ Spark Recordings in Isolated Mouse Ventricular Myocytes

Mouse ventricular myocytes were incubated with 5 µm Fluo‐4 AM in normal Tyrode's solution containing 1.8 mm Ca^2+^ for 15 min at room temperature. Cells were then washed with dye‐free normal Tyrode's solution for 30 min for de‐esterification and transferred to a chamber with a pair of parallel electrodes on a laser scanning confocal microscope (LSM 900, Carl Zeiss). After being paced at 1 Hz for at least 2 min and steady state Ca^2+^ transients were observed, pacing was stopped for 45 s and spontaneous Ca^2+^ sparks were counted.

### Compounds and Solutions

All the chemicals used in the electrophysiological experiments were purchased from Sigma‐Aldrich. Fluo‐4 AM and Fura‐2 AM were purchased from Invitrogen and stock solutions were both prepared in 1 mm 20% Pluronic F‐127 (Sangon Biotech, A600750) dissolved in DMSO (Sigma‐Aldrich, D2650). Isoproterenol was purchased from Sigma‐Aldrich (I6504) and stock solutions were prepared in 1 mm water. KN92 and KN93 were purchased from Selleck (S6507) and Abcam (Ab120980), respectively, and stock solutions were both prepared in 1 mm DMSO. The solutions of tetracaine (Sigma‐Aldrich, T7508) and caffeine (Sigma‐Aldrich, C0750) were freshly prepared when using. Flecainide was purchased from Sigma‐Aldrich (F6777) and stock solutions were prepared in 10 mm DMSO.

### Data Availability

The accession numbers for the RNA‐Seq data reported in this study were SUB14851162 and SUB11720381. The accession number for the ATAC‐Seq data reported in this study was SUB11731376. The mass spectrometry proteomics data were deposited to the ProteomeXchange Consortium (http://proteomecentral.proteomexchange.org) via the iProX partner repository with the dataset identifier PXD035912. All data needed to evaluate the conclusions in the paper were present in the paper and/or the Supporting Information. Additional data were available from authors upon request.

### Statistical Analysis

Statistical significance was determined by unpaired two‐tailed Student's *t*‐test to compare two groups and by one‐way ANOVA to compare multiple groups. A *p* value of <0.05 was considered statistically significant. ^*^ *p* < 0.05, ^**^ *p* < 0.01, ^***^ *p* < 0.001, and ^****^ *p* < 0.0001. Data were shown as mean ± standard error of the mean (SEM) and analyzed by GraphPad Prism (GraphPad Software).

## Conflict of Interest

The authors declare no conflict of interest.

## Author Contributions

J.S., X.W., H.F., and Y.S. contributed equally to this work. W.W., C.J., and P.L. designed and supervised the study. J.S., X.W., H.F., Y.S., T.G., H.Q., J.W., Z.P., Y.D., H.W., D.Z., T.Z., H.W., X.C., L.X., J.S., F.Y., Y.T., X.L., B.Y., and L.Z. performed the experiments and analyzed data. W.W. and P.L. wrote the paper.

## Supporting information



Supporting Information

## Data Availability

The data that support the findings of this study are available from the corresponding author upon reasonable request.
