# Supplementary material for: Decreased RYR2 Cluster Size and Abnormal SR Ca2+ Release Contribute to Arrhythmogenesis in TMEM43‐Related ARVC
Source: Adv Sci (Weinh). 2025 Sep 15;12(45):e12058. doi: 10.1002/advs.202512058 (PMC12677635; doi:10.1002/advs.202512058)
Supplement: Supplementary file 1 — Supporting Information [file ADVS-12-e12058-s001.pdf]

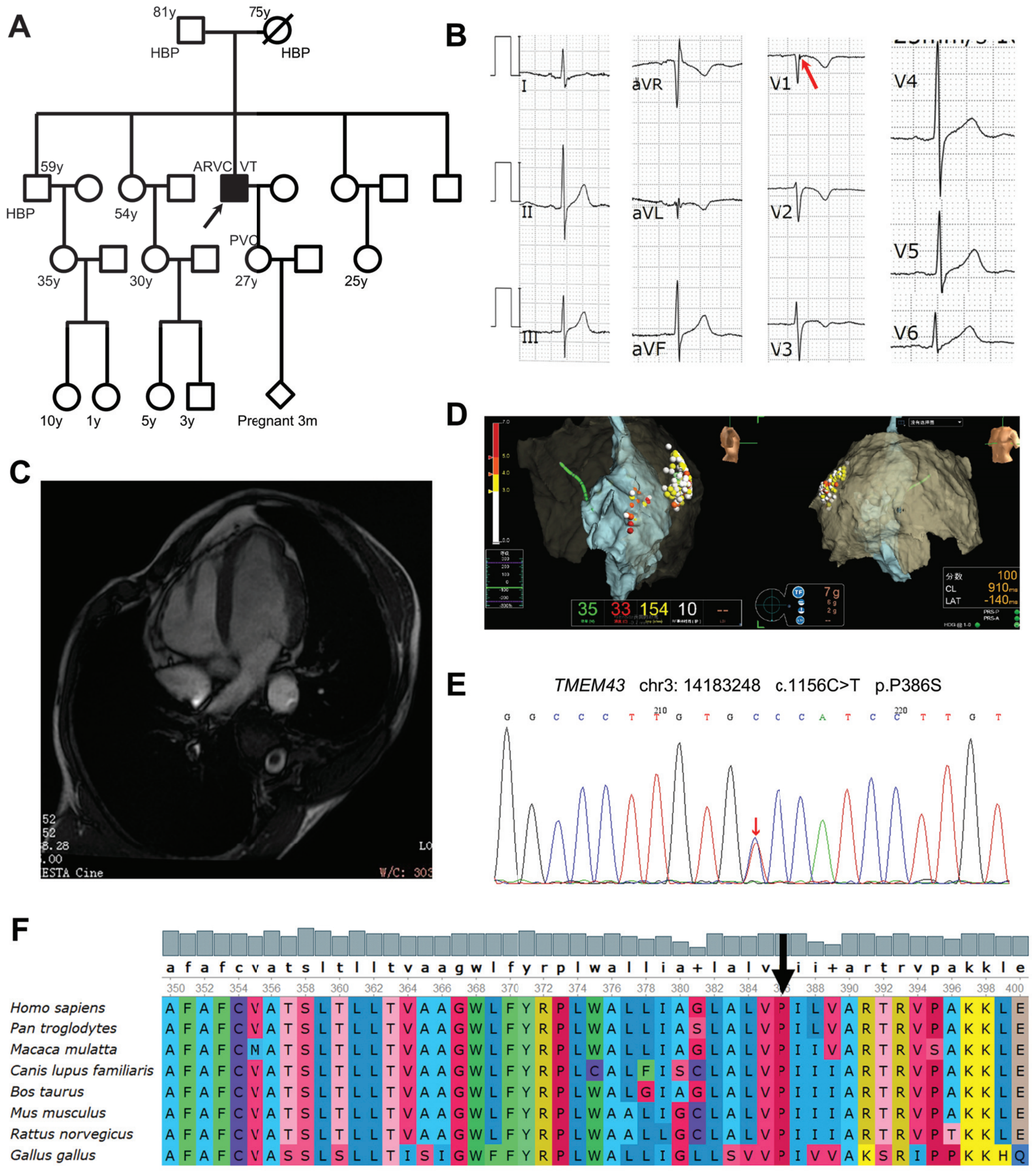

Figure S1. Clinical characteristics. A) The pedigree of the patient with ARVC recruited in this study. The black arrow indicates the proband. Females are denoted by circles and males are denoted by squares. Black squares indicate ventricular tachycardia (VT) phenotype. The death etiology of the mother of the proband is not clear. **B)** The representative 12-lead ECGs of the proband. The red arrow indicates typical episilon wave. **C)** Representative cardiac MRI plane of the proband. Fatty infiltration was detected in right ventricular wall. **D)** Ablation of VT by epicardium and endocardium access. Ablation sites mainly located in right ventricular outflow tract. **E)** Genetic testing identifying the missense mutation (P386S) of *TMEM43* in the proband. No mutations were found in other ARVC-associated genes (*PKP2*, *DSP*, *DSG2*, *DSC2*, *JUP*, *LMNA*, *DES*, *TTN*, *RYR2*, *SCN5A*, *PLN*, *TGFB3*). **F)** Amino acid sequence conservation of TMEM43 from various species in the region surrounding amino acid 386 (black arrow).

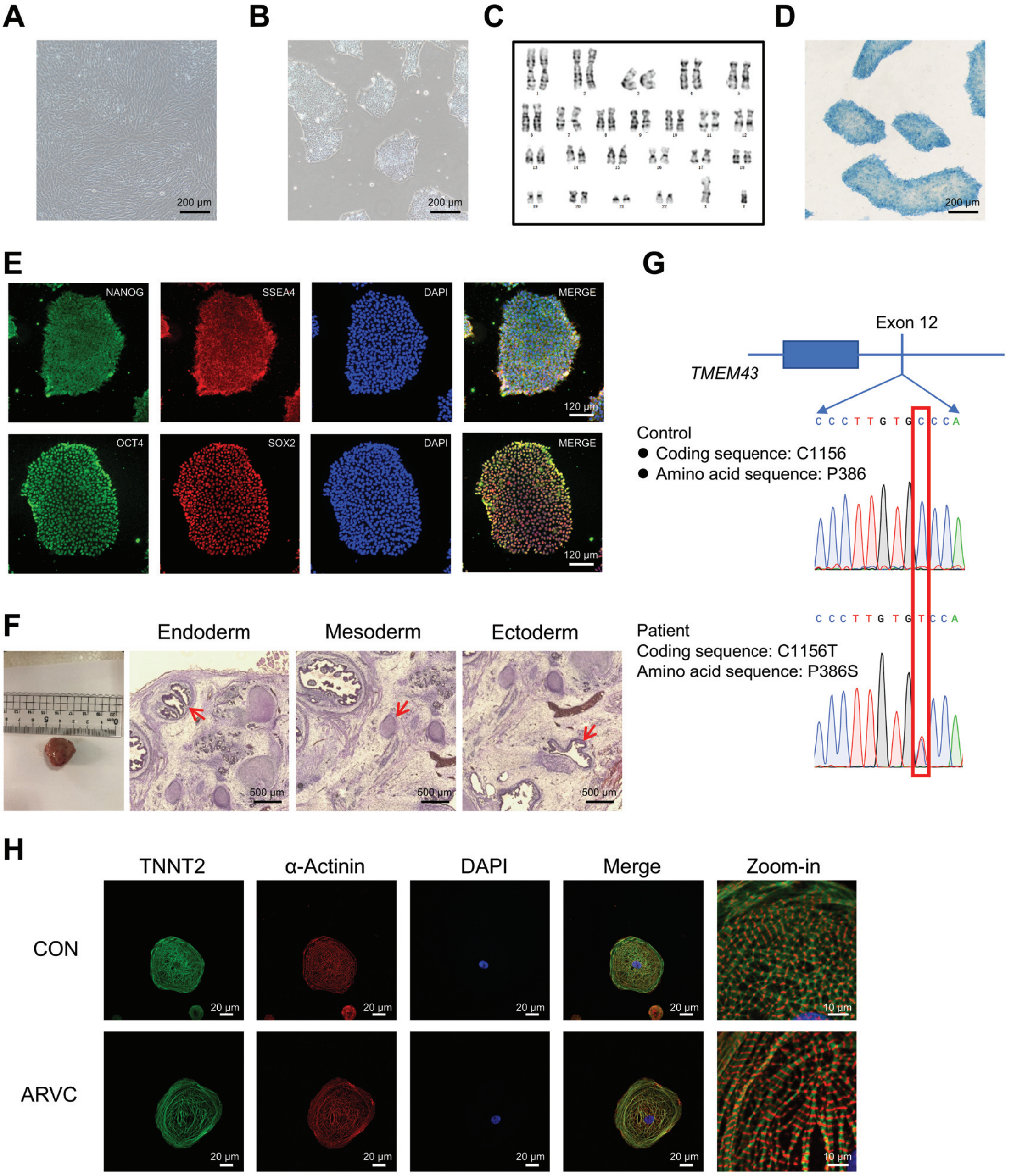

Figure S2. Generation and characterization of ARVC patient-specific iPSCs and iPSC-CMs carrying TMEM43-P386S. A, B) Typical morphology of skin fibroblasts and iPSCs derived from the ARVC patient. **C)** Karyotype of ARVC iPSCs. **D)** Alkaline Phosphatase (ALP) staining of ARVC iPSCs. **E)** Pluripotent staining of ARVC iPSCs using NANOG (green), SSEA4 (red), OCT4 (green) and SOX2 (red). DAPI indicates nuclear staining (blue). **F)** Teratoma formation assay using ARVC iPSCs showing derivations of three embryonic germ layers. **G)** Confirmation of existence of the *TMEM43* P386S mutation in ARVC iPSCs but not in control iPSCs. **H)** Representative graphs of cardiac-specific staining by TNNT2 (green) and α-actinin (red) in control and ARVC iPSC-CMs.

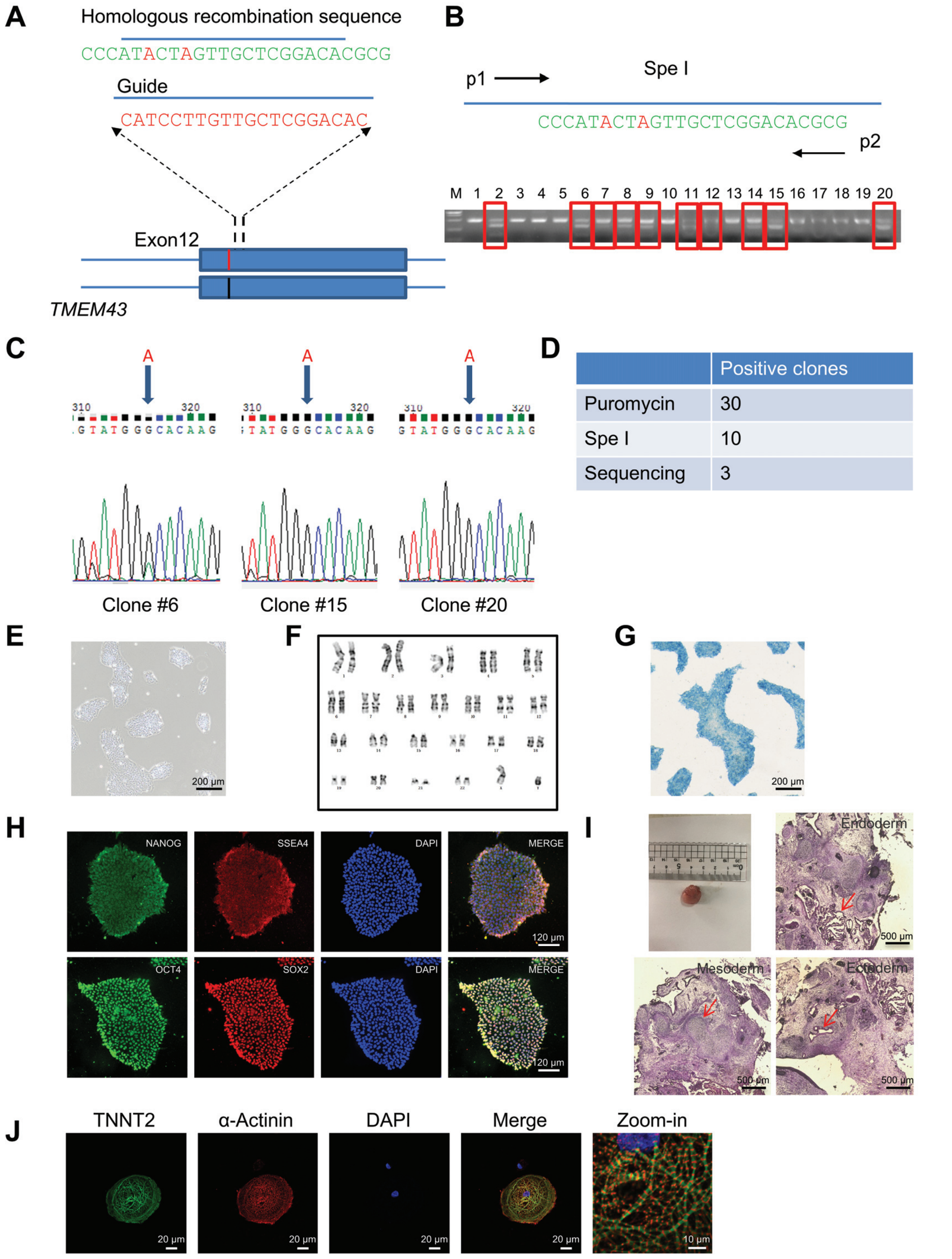

Figure S3. Gene correction of ARVC patient-specific iPSCs using CRISPR/Cas9. A) Strategy of correcting the *TMEM43* P386S mutation. The sequence of guide DNA (gDNA) is shown in red and homologous sequence is shown in green. **B)** Spe I restriction digestion of PCR products before and after gene correction. Enzyme digestion by Spe I identified 10 positive clones out of 30 puromycin-resistant clones indicated by red boxes. **C)** DNA sequencing demonstrates the correction of 1156C>T (P386S) mutation, and 3 gene-corrected clones were obtained (clone #6, clone #15 and clone #20). **D)** Summary of number of positive clones after puromycin screening (30

positive clones), enzyme digestion by Spe I (10 positive clones) and DNA sequencing (3 positive clones). **E)** Typical morphology of GC iPSCs. **F)** Karyotype of GC iPSCs. **G)** ALP staining of ARVC iPSCs. **H)** Pluripotent staining of GC iPSCs using NANOG (green), SSEA4 (red), OCT4 (green) and SOX2 (red). DAPI indicates nuclear staining (blue). **I)** Teratoma formation assay using GC iPSCs showing derivations of three embryonic germ layers. **J)** Representative graphs of cardiac-specific staining by TNNT2 (green) and α-actinin (red) in GC iPSC-CMs.

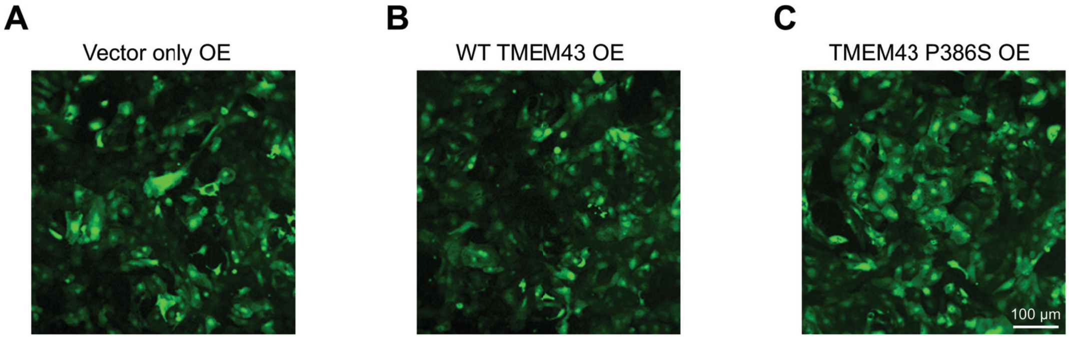

Figure S4. Validation of the transfection efficiency. A-C) Representative graphs showing the transfection efficiency after lentiviral infection in control iPSC-CMs overexpressing lentiviral-GFP (Vector only OE), lentiviral-WT TMEM43-GFP (WT TMEM43 OE) and lentiviral-TMEM43 P386S-GFP (TMEM43 P386S OE), respectively. n= 5-6 independent experiments.

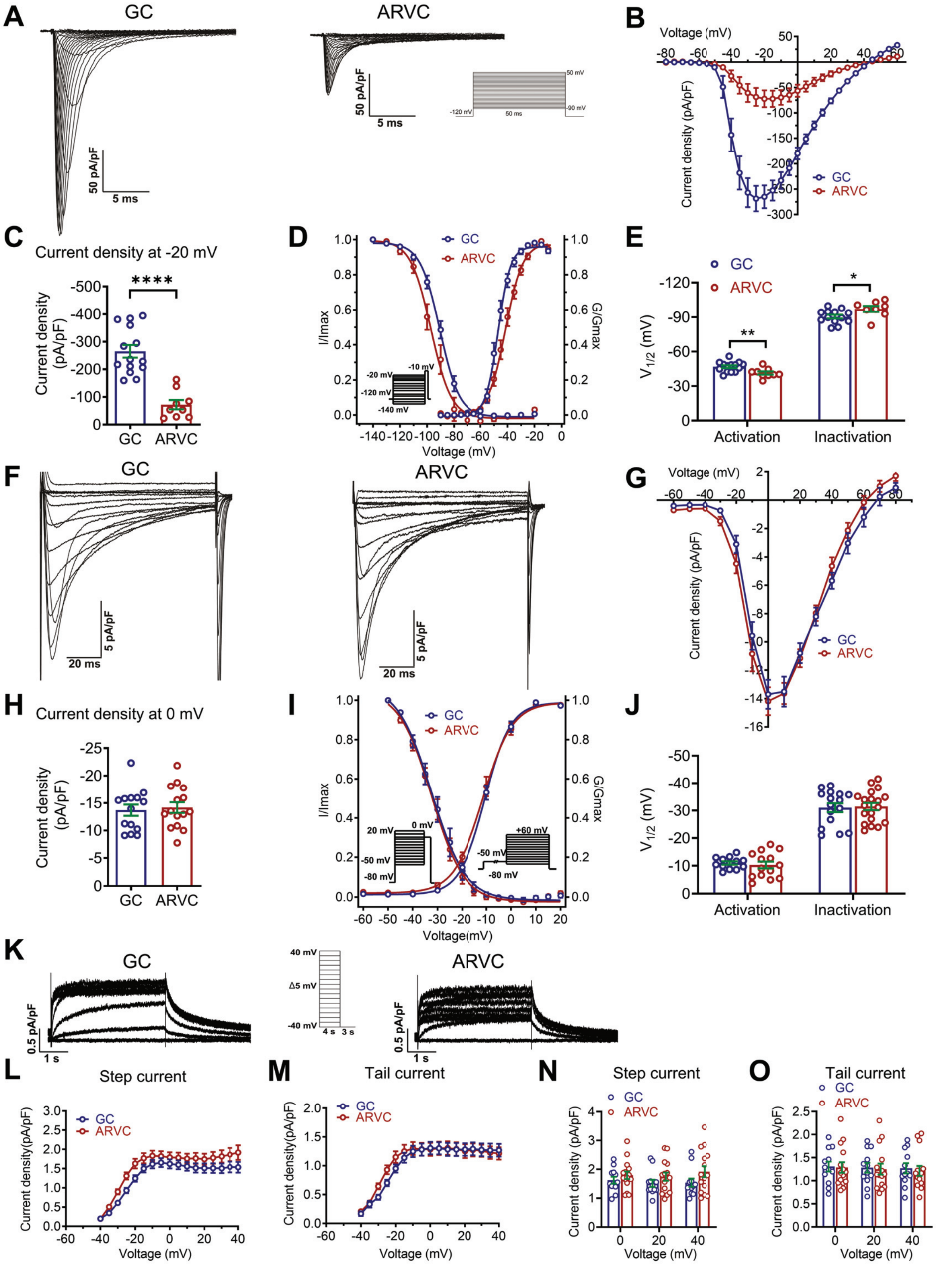

Figure S5. Isolation of endogenous sodium, L-type calcium and potassium currents from GC and ARVC iPSC-CMs by patch clamp. A) Representative sodium current traces recorded in GC and ARVC iPSC-CMs. **B)** Curve of sodium current–voltage relationship between GC and ARVC iPSC-CMs. **C)** Bar graph to compare the peak sodium current density at -20 mV between GC and ARVC iPSC-CMs. n= 9-14 cells in 2 different iPSC lines. **D)** Steady-state activation and inactivation of sodium currents in GC and ARVC iPSC-CMs. The dotted lines represent fitted data calculated by a Boltzmann function. **E)** Bar graph to compare the $V_{1/2}$ of steady-state activation or

inactivation of sodium currents between GC and ARVC iPSC-CMs. n= 8-14 cells in 2 different iPSC lines. **F)** Representative L-type calcium current traces recorded in GC and ARVC iPSC-CMs. **G)** Curve of L-type calcium current–voltage relationship between GC and ARVC iPSC-CMs. **H)** Bar graph to compare the peak L-type calcium current density at 0 mV between GC and ARVC iPSC-CMs. n= 14-15 cells in 2 different iPSC lines. **I)** Steady-state activation and inactivation of L-type calcium currents in GC and ARVC iPSC-CMs. The dotted lines represent fitted data calculated by a Boltzmann function. **J)** Bar graph to compare the $V_{1/2}$ of steady-state activation or inactivation of L-type calcium currents between GC and ARVC iPSC-CMs. n= 13-19 cells in 2 different iPSC lines. **K)** Representative total potassium current traces recorded in GC and ARVC iPSC-CMs. **L, M)** Curve of potassium current–voltage relationship between GC and ARVC iPSC-CMs. **N, O)** Bar graphs to compare the potassium current amplitude at 0, 20 and 40 mV between GC and ARVC iPSC-CMs. n= 12-15 cells in 2 different iPSC lines. The current amplitude was normalized to the cell capacitance to obtain current density value (pA/pF). For all panels, data are represented as mean ± SEM. $^{*}p< 0.05$; $^{**}p< 0.01$; $^{****}p< 0.0001$, unpaired two-tailed Student's *t* test (**C, E, H, J, N, O**).

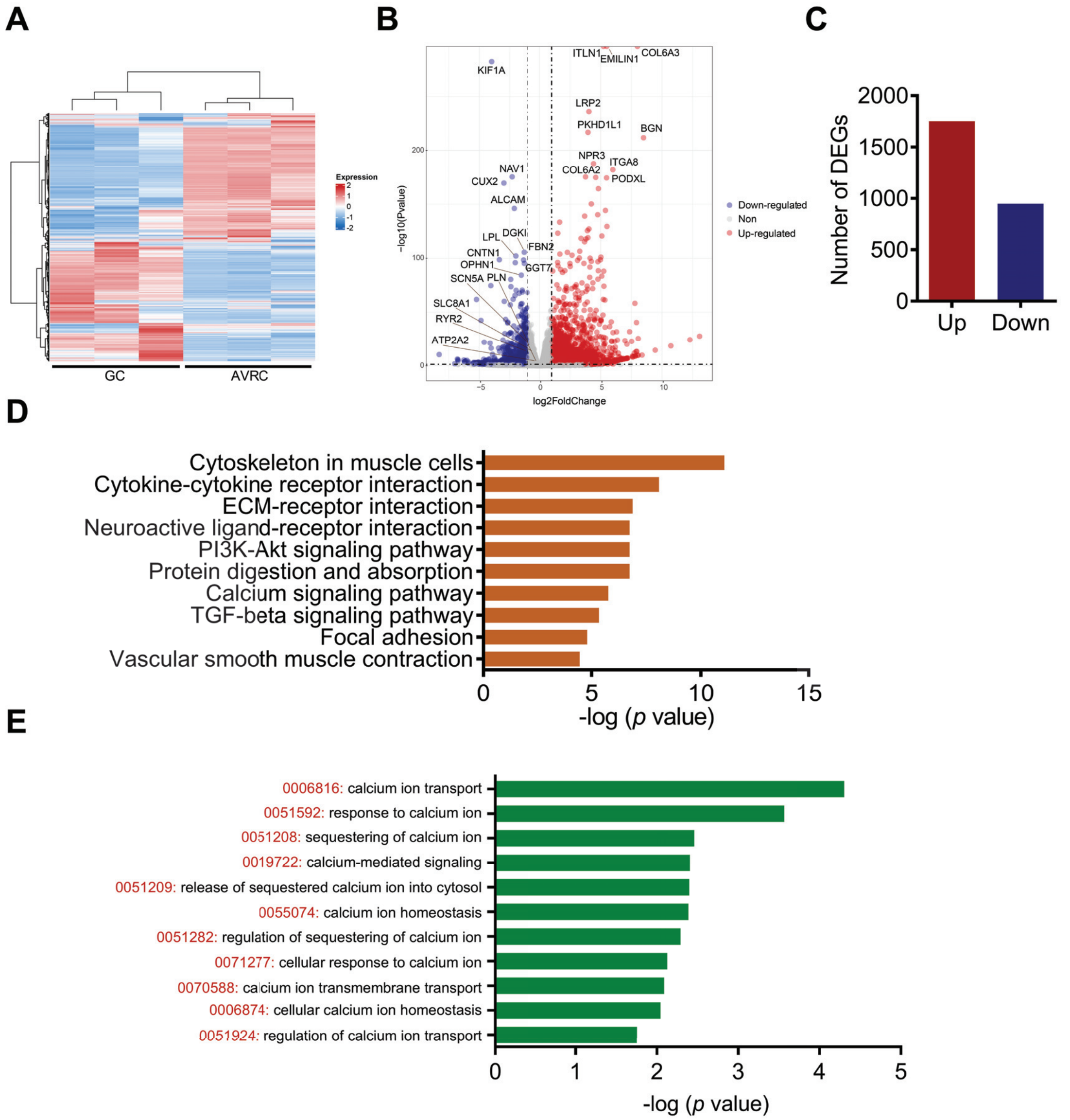

Figure S6. Transcriptomic analysis of ARVC iPSC-CMs. A) Heatmap demonstrating the differential gene expression pattern between GC and ARVC iPSC-CMs. **B)** Volcano plot of differential expressed genes (DEGs). Red points represent up-regulated DEGs; blue points represent down-regulated DEGs; grey points represent non-DEGs. **C)** Bar graph to show up- and down-regulated DEGs. **D)** Kyoto Encyclopedia of Genes and Genomes (KEGG) enrichment analysis. **E)** Gene ontology (GO) enrichment analysis.

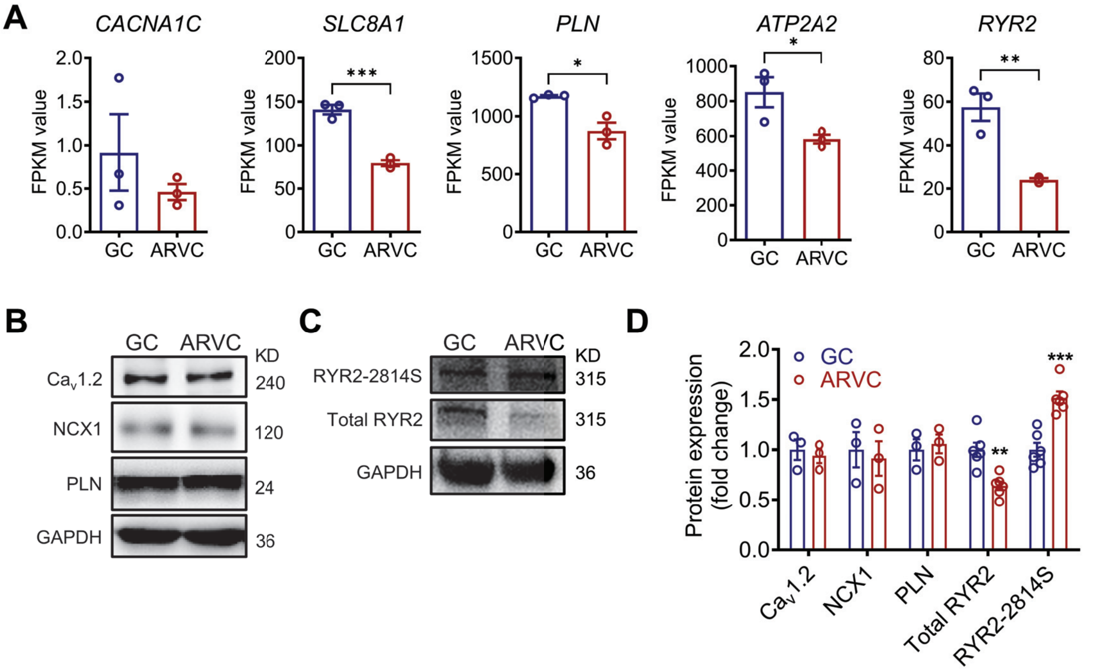

Figure S7. Transcript and protein expression levels of key Ca^{2+}-handling genes in GC and ARVC iPSC-CMs. A) Bar graphs to compare the FPKM values of *CACNA1C* (encoding $Ca_V1.2$), *SLC8A1* (encoding NCX1), *PLN* (encoding PLN), *ATP2A2* (encoding SERCA2a) and *RYR2* (encoding RYR2) between GC and ARVC iPSC-CMs by RNA-Seq. n= 3 batches of iPSC-CMs from independent differentiations. **B, C)** Western blot analysis of expression of $Ca_V1.2$, NCX1, PLN and RYR2 in GC and ARVC iPSC-CMs, respectively. RYR2-2814S denotes phosphorylated RYR2 at Ser2814. **D)** Bar graph to compare the expression levels of key Ca^{2+}-handling proteins between GC and ARVC iPSC-CMs. n= 3-6 batches of iPSC-CMs from independent differentiations. For all panels, data are represented as mean ± SEM. $^{*}p < 0.05$; $^{**}p < 0.01$; $^{***}p < 0.001$, unpaired two-tailed Student's *t* test (**D**).

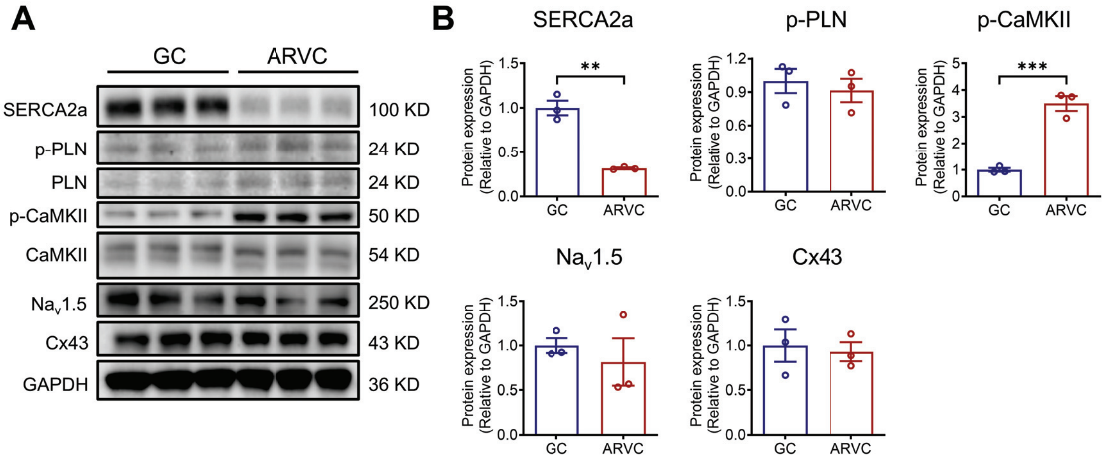

Figure S8. Comparison of protein expression of SERCA2a, PLN, CaMKII, Na_v1.5 and Cx43 between GC and ARVC iPSC-CMs. A) Western blot analysis of expression of SERCA2a, phosphorylated PLN (Ser16/Thr17), phosphorylated CaMKII (phospho T286), Na_v1.5 and Cx43 in GC and ARVC iPSC-CMs, respectively. p-PLN and p-CaMKII denote phosphorylated PLN and CaMKII. **B)** Bar graphs to compare the protein expression levels in A. n= 3 batches of iPSC-CMs from independent differentiations. For all panels, data are represented as mean ± SEM. $^{**}p< 0.01$; $^{***}p< 0.001$, unpaired two-tailed Student's *t* test (**B**).

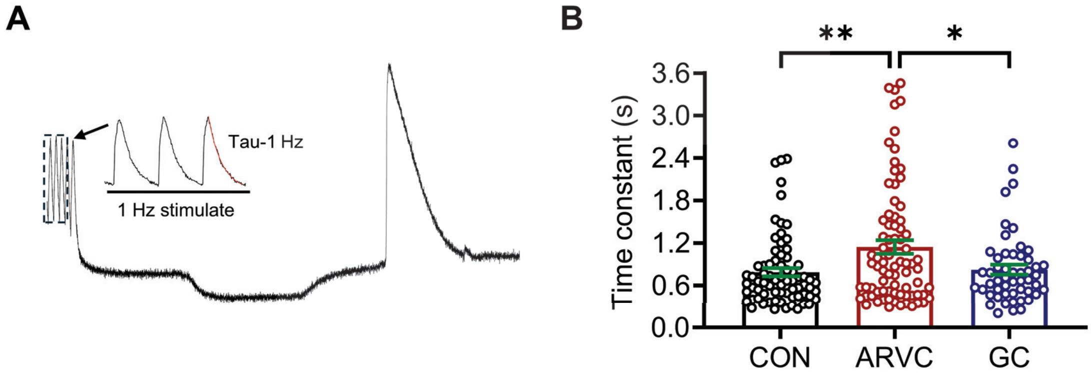

Figure S9. Assessment of SR Ca^{2+} reserve function. A) Illustration of the mono-exponential curve fitting for Ca^{2+} transient decay at 1 Hz stimulation (Tau-1 Hz). **B)** Bar graph to compare the time constant of Ca^{2+} transient decay (Tau-1 Hz) between control, ARVC and GC iPSC-CMs. n= 52-78 cells in 2 different iPSC lines. For all panels, data are represented as mean ± SEM. $^{*}p < 0.05$; $^{**}p < 0.01$, one-way ANOVA followed by Tukey's HSD (honestly significant difference) post-hoc test (**B**).

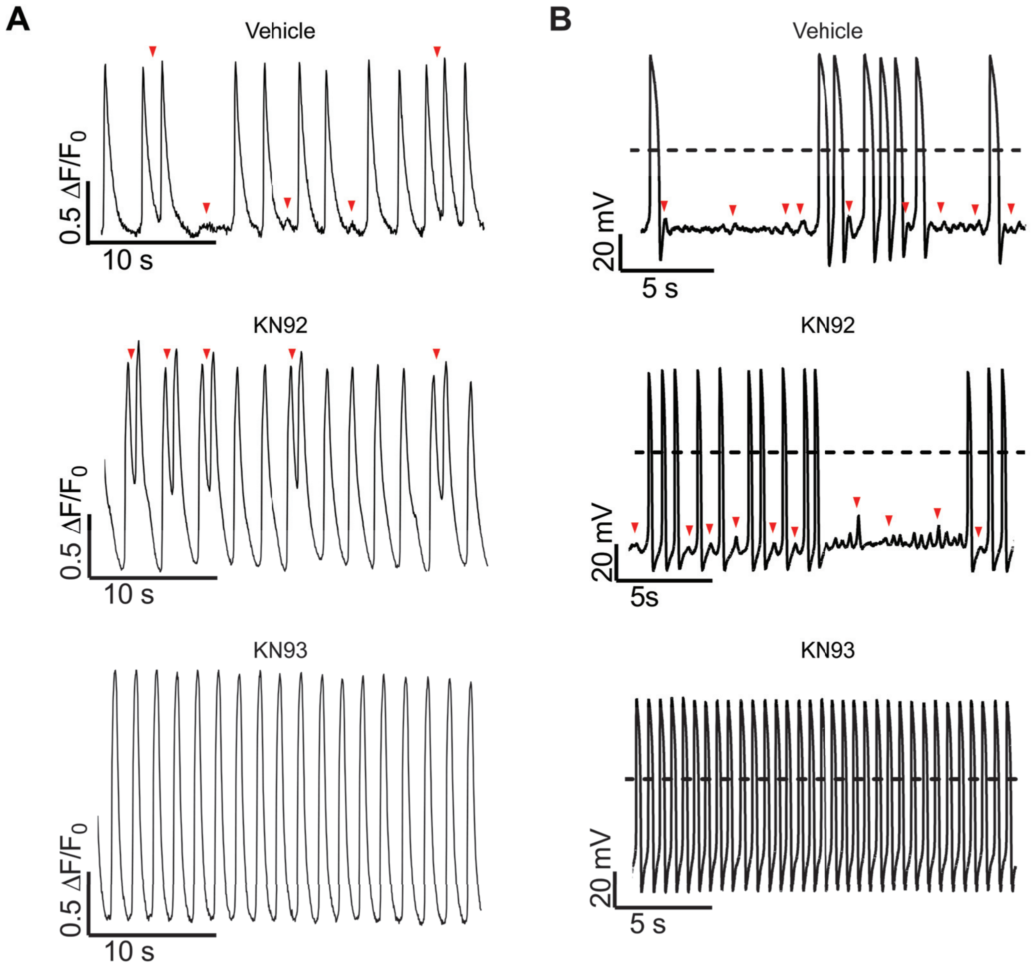

Figure S10. Anti-arrhythmic effect of KN93 on ARVC iPSC-CMs. A) Representative Ca^{2+} transient tracings recorded by Fluo-4 Ca^{2+} imaging from ARVC iPSC-CMs treated with DMSO (vehicle), KN92 (the inactive analogue) or KN93 (CaMKII-specific inhibitor), respectively. Red arrows indicate the irregular arrhythmia-like Ca^{2+} transients. **B)** Representative action potential tracings recorded by single-cell patch clamp from ARVC iPSC-CMs treated with DMSO (vehicle), KN92 or KN93, respectively. Dash lines indicate 0 mV. Red arrows indicate the arrhythmias.

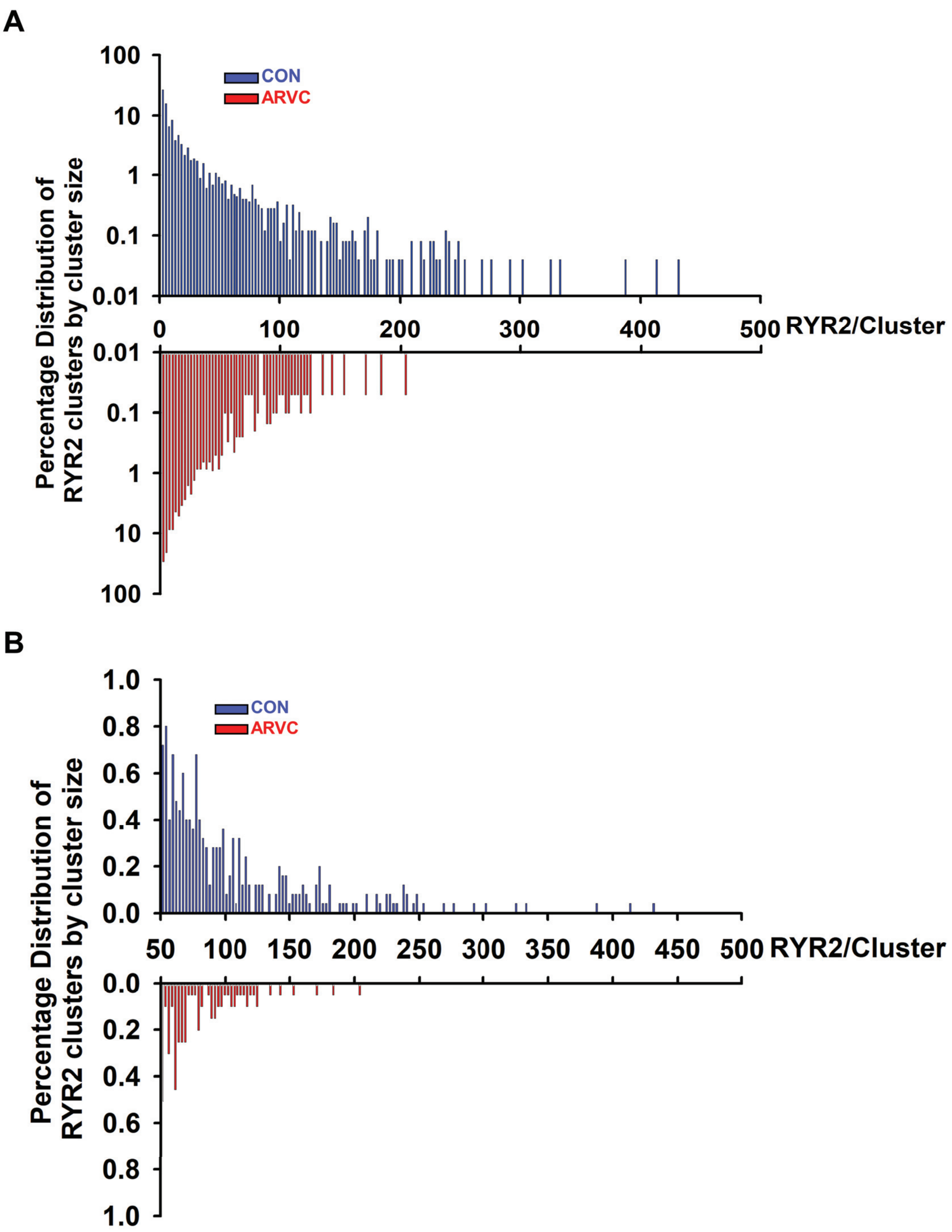

Figure S11. Influenced nanoscale organization of RYR2 clusters in ARVC iPSC-CMs. A) The distribution of the number of RYR2 clusters across increasing cluster size on a logarithmic scale. **B)** The distribution of the number of RYR2 clusters across increasing cluster size (for clusters with 50 or more RYR2s only).

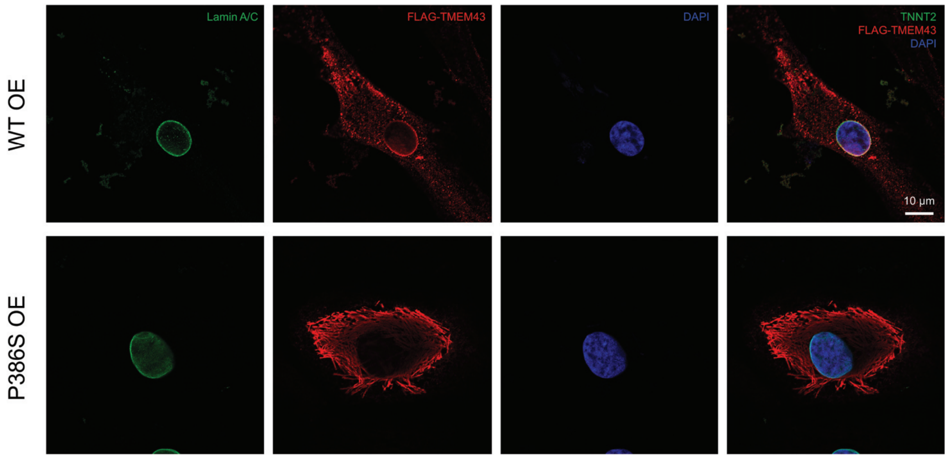

Figure S12. Subcellular translocalization of TMEM43 in P386S iPSC-CMs. Representative graphs of co-staining by lamin A/C (green) and FLAG-TMEM43 (red) in control iPSC-CMs overexpressing lentiviral-WT TMEM43-GFP (WT OE) and lentiviral-TMEM43 P386S-GFP (P386S OE). DAPI indicates nuclear staining (blue). n= 18-20 views in 3 independent experiments.

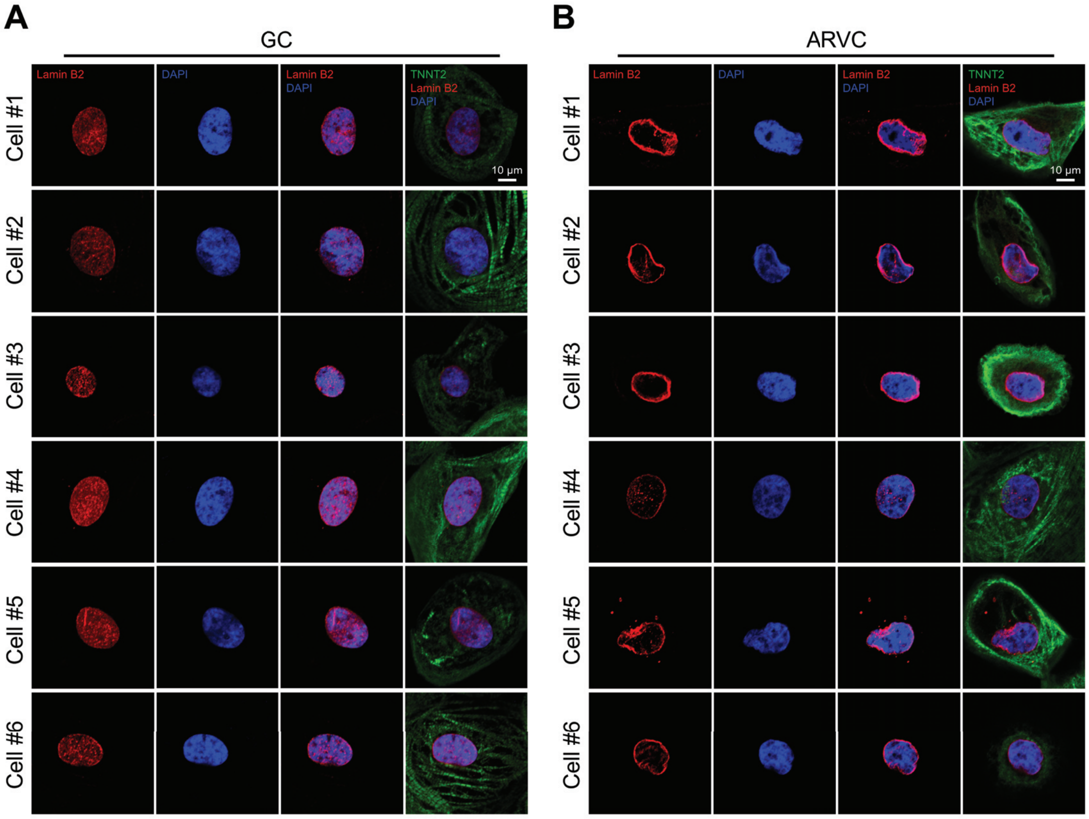

Figure S13. Analysis of lamin B2 localization in GC and ARVC iPSC-CMs. A, B) Representative graphs of co-staining by TNNT2 (green) and lamin B2 (red) in GC and ARVC iPSC-CMs. DAPI indicates nuclear staining (blue). n= 27-33 views in 3 independent experiments. Data were collected from 2 different iPSC lines.

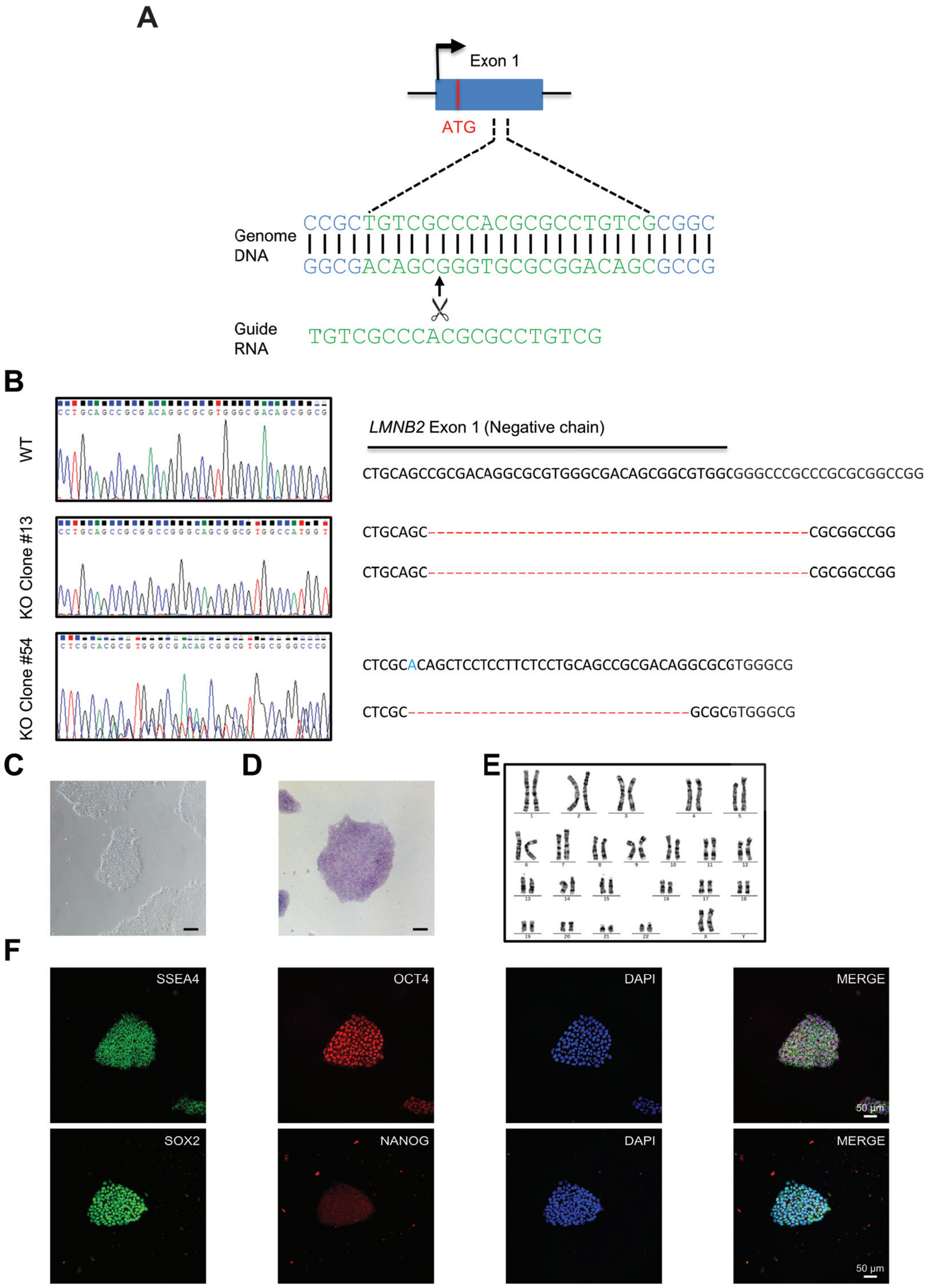

Figure S14. Generation and characterization of lamin B2 knockout iPSCs using CRISPR/Cas9. A) Schematic of Cas9/gRNA-targeting sites in *LMNB2* (encoding Lamin B2). **B)** Sanger sequencing of control iPSCs (WT) and Lamin B2 knockout (KO) iPSCs (Clone#13 and Clone#54). **C)** Typical morphology of lamin B2 KO iPSCs. **D)** ALP staining of lamin B2 KO iPSCs. **E)** Karyotype of lamin B2 KO iPSCs. **F)** Pluripotent staining of lamin B2 KO iPSCs using SSEA4 (green), OCT4 (red), SOX2 (green) and NANOG (red). DAPI indicates nuclear staining (blue).

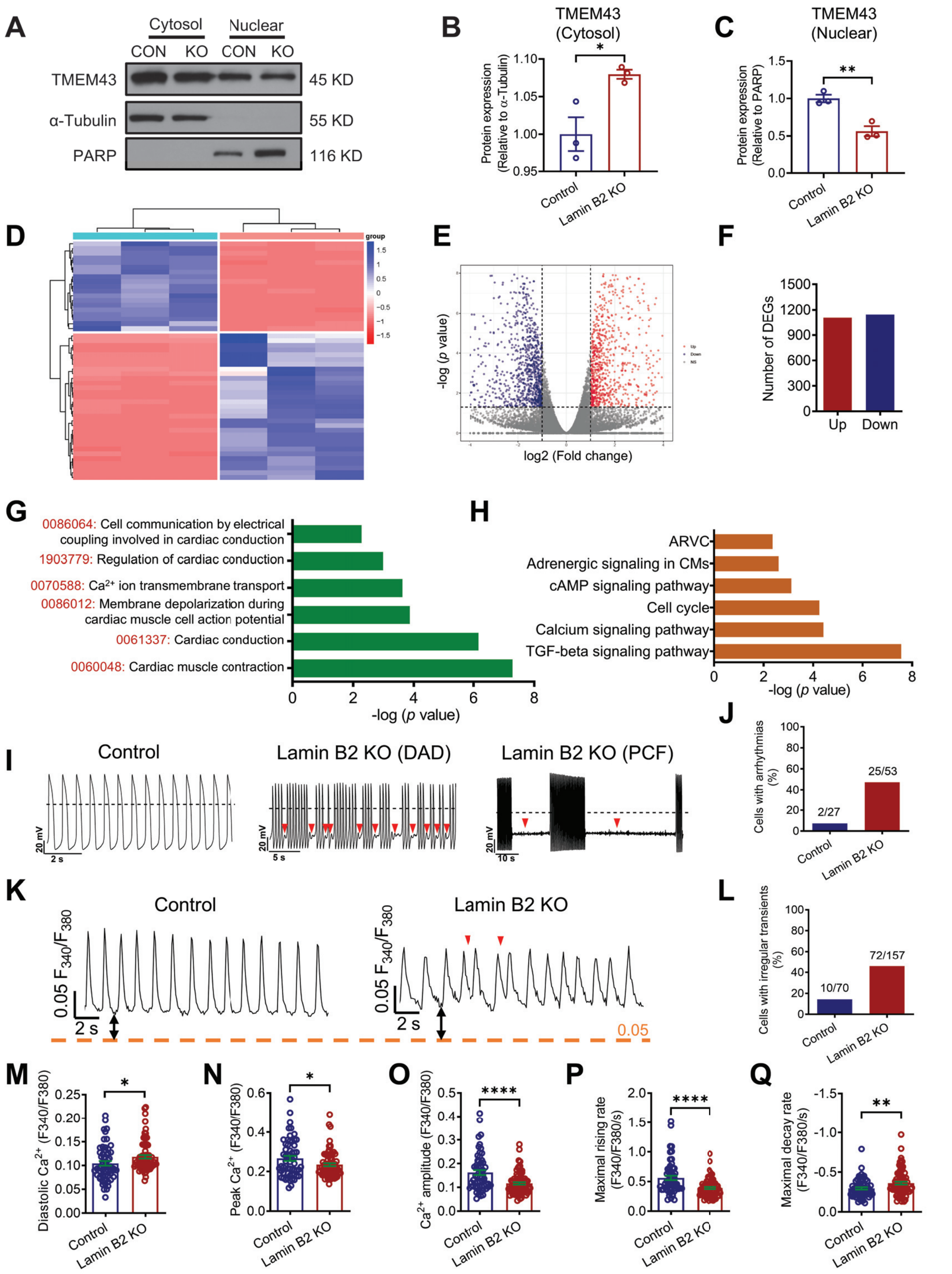

Figure S15. Lamin B2 depletion recapitulates arrhythmic phenotype of P386S iPSC-CMs. A) Western blot analysis of protein expression of cytosol and nuclear TMEM43 in control and lamin B2 KO iPSC-CMs. **B, C)** Bar graphs to compare the protein expression of cytosol and nuclear TMEM43 between control and lamin B2 KO iPSC-CMs. n= 3 batches of iPSC-CMs from independent differentiations. **D)** Heatmap demonstrating the differential gene expression pattern between control and lamin B2 KO iPSC-CMs. **E)** Volcano plot of DEGs. Red points represent up-regulated DEGs; blue points represent down-regulated DEGs; grey points represent non-DEGs. **F)** Bar

graph to show up- and down-regulated DEGs. **G)** GO enrichment analysis. **H)** KEGG enrichment analysis. **I)** Representative action potential tracings recorded by single-cell patch clamp from control and lamin B2 KO iPSC-CMs. Dash lines indicate 0 mV. Red arrows indicate the delayed afterdepolarization (DAD) or paroxysmal cellular flutter (PCF) arrhythmias. **J)** Bar graph to compare the percentage of cells with arrhythmias between control (n= 27) and lamin B2 KO (n= 53) iPSC-CMs. Data were collected from 2 different iPSC lines. **K)** Representative Ca^{2+} transient tracings recorded by Fura-2 Ca^{2+} imaging from control and lamin B2 KO iPSC-CMs. Red arrows indicate the irregular Ca^{2+} transients. **L)** Bar graph to compare the percentage of cells with arrhythmic transients between control (n= 70) and lamin B2 KO (n= 157) iPSC-CMs. Data were collected from 2 different iPSC lines. **M-Q)** Bar graphs to compare key Ca^{2+} transient parameters between control and lamin B2 KO iPSC-CMs, including diastolic Ca^{2+}, peak Ca^{2+}, Ca^{2+} amplitude, maximal rising rate and maximal decay rate. n= 58-76 cells in 2 different iPSC lines. For all panels, data are represented as mean ± SEM. $^{*}p< 0.05$; $^{**}p< 0.01$; $^{****}p< 0.0001$, unpaired two-tailed Student's *t* test (**B, C, M-Q**).

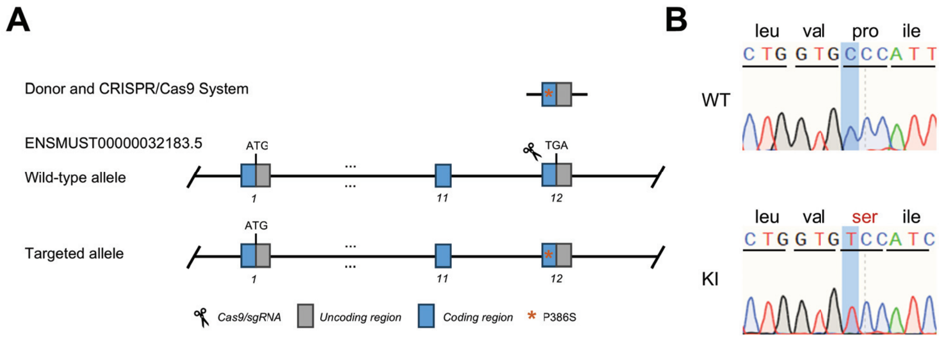

Figure S16. Generation of the TMEM43-P386S KI mice model. A) Schematic of the targeting vector and generation of TMEM43-P386S KI mice. **B)** DNA sequencing confirming the successful generation of KI mice carrying the P386S mutation.

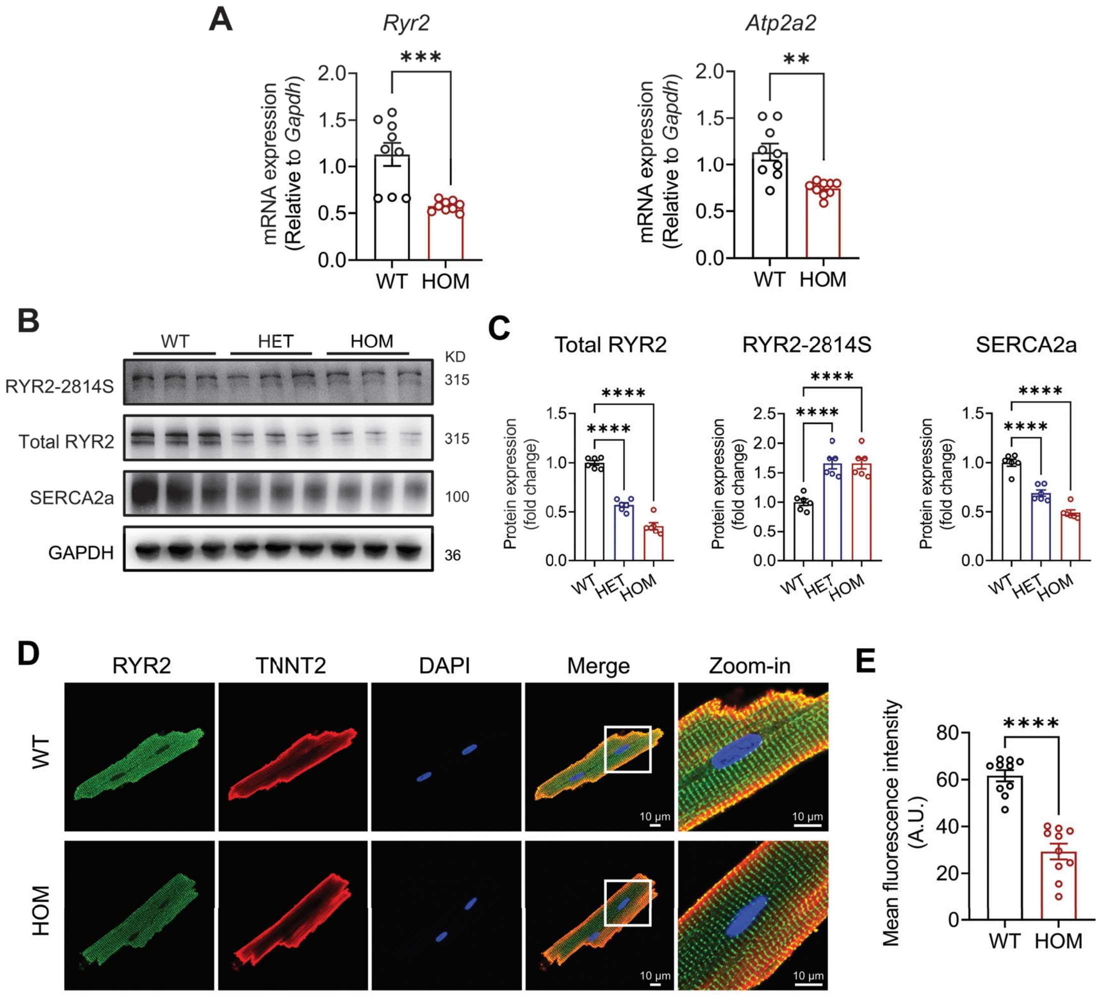

Figure S17. Reduced RYR2 and SERCA2a expression in the TMEM43-P386S KI mouse hearts. A) Bar graphs to compare the mRNA expression of *Ryr2* and *Atp2a2* between heart tissues collected from WT and HOM KI mice. n= 9 mouse hearts. **B, C)** Western blot analysis of protein expression of RYR2 and SERCA2a in heart tissues collected from WT, HET, and HOM KI mice. RYR2-2814S denotes phosphorylated RYR2 at Ser2814. n= 6 mouse hearts. The mice used were all 4 months old, and the collected heart tissues were specifically from the ventricles. **D)** Representative graphs of co-staining by RYR2 (green) and TNNT2 (red) in WT and HOM KI ventricular myocytes. DAPI indicates nuclear staining (blue). Ventricular myocytes were isolated from 4-month-old mice. **E)** Bar graph to compare the mean fluorescence intensity of RYR2 between the two groups in D. n= 10 cells per group from 2 hearts/genotype. For all panels, data are represented as mean ± SEM. $^{**}p < 0.01$; $^{***}p < 0.001$; $^{****}p < 0.0001$, unpaired two-tailed Student's t test (**A, E**) or one-way ANOVA with Dunnett's multiple comparison test versus control (WT mouse hearts) (**C**).

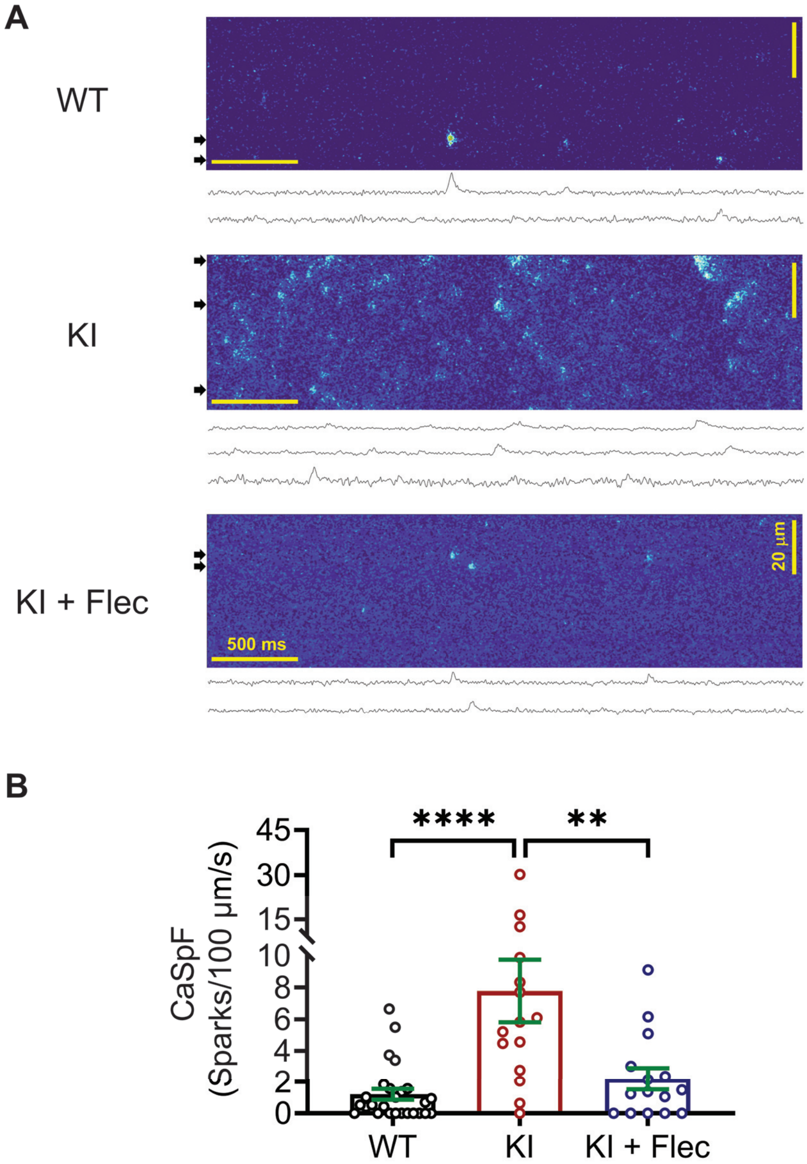

Figure S18. Flecainide suppresses the increased Ca^{2+} spark frequency in the TMEM43-P386S KI mouse cardiomyocytes. A) Representative recordings of Ca^{2+} sparks from WT ventricular myocytes treated with DMSO, HOM KI ventricular myocytes treated with DMSO, and HOM KI ventricular myocytes treated with 10 µM flecainide (Flec). Ventricular myocytes were isolated from 4-month-old mice. **B)** Bar graph to compare the Ca^{2+} spark frequency (CaSpF) between different groups in A. n= 15-26 cells per group from 3-4 hearts/genotype. For all panels, data are represented as mean ± SEM. $^{**}p< 0.01$; $^{****}p< 0.0001$, one-way ANOVA followed by Tukey's HSD post-hoc test (**B**).

Table S1. Clinical information of the healthy control subjects and the ARVC patient recruited in this study.

	Age	Gender	Ethnicity	History of heart disease
**CON #1**	37	Female	Han Chinese	No history of heart disease. ECG, echo revealed no structure disease. Stress test showed negative results.
**CON #2**	53	Male	Han Chinese	No history of heart disease. ECG, echo revealed no structure disease. Stress test showed negative results.
**ARVC**	54	Male	Han Chinese	The patient presented with paroxysmal palpitation and syncope in March 2016. The ECG in emergency room showed ventricular tachycardia (VT) of left bundle branch block morphology. The rate of VT was 176 bpm. Cardioversion was applied to terminate the VT. The VT recurred twice in April 2016 and May 2016.

Table S2. Summary of action potential parameters in control, ARVC, GC and KI ventricular-like iPSC-CMs.

	Beating rate (beats/min)	MDP (mV)	Overshoot (mV)	APA (mV)	APD_{50} (ms)	APD_{90} (ms)	V_{max} (V/s)
**CON1**	73.9 ± 4.9	-62.0 ± 0.9	49.2 ± 1.1	111.2 ± 1.4	279.8 ± 9.4	322.5 ± 10.2	17.8 ± 1.0
**CON2**	75.0 ± 3.5	-63.0 ± 0.6	47.6 ± 1.2	110.7 ± 1.0	263.1 ± 12.8	301.4 ± 12.7	15.8 ± 0.9
**ARVC**	78.7 ± 4.3	-56.6 ± 0.8	47.1 ± 1.1	103.5 ± 1.1	239.7 ± 14.0	287.3 ± 14.6	12.7 ± 0.7
**GC**	70.5 ± 4.2	-65.1 ± 0.7	45.3 ± 0.7	110.4 ± 1.0	252.8 ± 18.8	303.1 ± 21.6	14.3 ± 0.8
**KI**	72.8 ± 4.7	-61.9 ± 0.6	51.4 ± 0.8	113.4 ± 1.0	283.9 ± 12.2	321. 5± 13.2	12.9 ± 0.4

Table S3. Mass spectrometry identification results of targeted proteins.

	Protein	Lamin B2	PKP2	JUP	Lamin A/C	Lamin B1
CON	Score	6.2151	5.1013	9.3661	51.122	31.271
	Peptides	5	2	5	13	9
	Sequence coverage (%)	7.1	2	11.7	21.7	18.4
	LFQ intensity	7,460,600	15,340,000	9,486,000	57,893,000	4,777,500
ARVC	Score	6.2151	5.1013	9.3661	51.122	31.271
	Peptides	7	2	4	10	14
	Sequence coverage (%)	8.7	2	7.4	16.6	25.6
	LFQ intensity	11,862,000	12,061,000	12,300,000	21,723,000	12,539,000
GC	Score	6.2151	5.1013	9.3661	51.122	31.271
	Peptides	8	2	5	17	15
	Sequence coverage (%)	9.5	2	11.7	27.7	26.5
	LFQ intensity	9,031,000	15,973,000	13,921,000	59,168,000	18,788,000

Table S4. Summary of action potential parameters in control and Lamin B2 KO ventricular-like iPSC-CMs.

	**Beating rate (beats/min)**	**MDP (mV)**	**Overshoot (mV)**	**APA (mV)**	**APD_{50} (ms)**	**APD_{90} (ms)**	**V_{max} (V/s)**
**Control**	84.6 ± 6.6	-63.3 ± 0.8	45.3 ± 0.8	108.7 ± 0.9	231.2 ± 9.7	280.3 ± 11.3	15.6 ± 1.0
**Lamin B2 KO**	97.9 ± 4.9	-55.0 ± 1.1	48.5 ± 1.1	103.5 ± 1.6	245.8 ± 16.3	287.1 ± 17.5	13.5 ± 0.6
